# Mediator complex subunit 1 promotes oral squamous cell carcinoma progression by activating MMP9 transcription and suppressing CD8^+^ T cell antitumor immunity

**DOI:** 10.1186/s13046-024-03191-9

**Published:** 2024-09-30

**Authors:** Zhe Li, Mengke Sun, Ruimeng Yang, Zheng Wang, Qianyu Zhu, Yue Zhang, Haosun Yang, Zhaosong Meng, Lizhi Hu, Lei Sui

**Affiliations:** 1 Department of Prosthodontics, Tianjin Medical University School and Hospital of Stomatology, Tianjin Key Laboratory of Oral Soft and Hard Tissues Restoration and Regeneration, Tianjin Medical University Institute of Stomatology, 12 Qixiangtai Road, Tianjin, 300070 China; 2 Immunology Department, Key Laboratory of Immune Microenvironment and Disease (Ministry of Education), Tianjin Medical University, 22 Qixiangtai Road, Tianjin, 300070 China; 3 Department of Oral and Maxillofacial Surgery, Tianjin Medical University School and Hospital of Stomatology, Tianjin Key Laboratory of Oral Soft and Hard Tissues Restoration and Regeneration, Tianjin Medical University Institue of Stomatology, 12 Qixiangtai Road, Tianjin, 300070 China

**Keywords:** The Mediator complex subunit 1, Matrix metalloprotein 9, Transcriptional regulation, Programmed death-ligand 1, Notch signaling pathway, Oral squamous cell carcinoma

## Abstract

**Background:**

The role of Mediator complex subunit 1 (MED1), a pivotal transcriptional coactivator implicated in diverse biological pathways, remains unexplored in the context of oral squamous cell carcinoma (OSCC). This study aims to elucidate the contributory mechanisms and potential impact of MED1 on the progression of OSCC.

**Methods:**

The expression and clinical significance of MED1 in OSCC tissues were evaluated through the bioinformatics analyses. The effects of MED1 on the biological behavior of OSCC cancer cells were assessed both in vitro and in vivo. Dual-luciferase reporter assay, chromatin immunoprecipitation (ChIP) assay, bioinformatic analysis, CD8^+^ T cell isolation experiment, coculture experiment, enzyme-linked immunosorbent assay (ELISA), and flow cytometric analysis were employed to elucidate the underlying mechanism through which MED1 operates in the progression of OSCC.

**Results:**

MED1 exhibited upregulation in both OSCC tissues and multiple OSCC cell lines, which correlated with decreased overall survival in patients. In vitro experiments demonstrated that knockdown of MED1 in metastatic OSCC cell lines SCC-9 and UPCI-SCC-154 hindered cell migration and invasion, while overexpression of MED1 promoted these processes. Whereas, MED1 knockdown had no impact on proliferation of cell lines mentioned above. In vivo studies further revealed that downregulation of MED1 effectively suppressed distant metastasis in OSCC. Mechanistically, MED1 enhanced the binding of transcription factors c-Jun and c-Fos to the matrix metalloprotein 9 (MMP9) promoters, resulting in a significant upregulation of MMP9 transcription. This process contributes to the migration and invasion of SCC-9 and UPCI-SCC-154 cells. Furthermore, MED1 modulated the expression of programmed death-ligand 1 (PD-L1) through the Notch signaling pathway, consequently impacting the tumor-killing capacity of CD8^+^ T cells in the tumor microenvironment.

**Conclusions:**

Our findings indicate that MED1 plays a pivotal role in OSCC progression through the activation of MMP9 transcription and suppression of CD8^+^ T cell antitumor immunity, suggesting that MED1 may serve as a novel prognostic marker and therapeutic target in OSCC.

**Supplementary Information:**

The online version contains supplementary material available at 10.1186/s13046-024-03191-9.

## Introduction

Ranked as the eighth most prevalent form of cancer globally, oral squamous cell carcinoma (OSCC) stands as the predominant type of head and neck cancer [1]. OSCC specifically denotes squamous cell carcinomas originating within the oral cavity, encompassing the cheeks, lips, mouth floor, tongue, and palate. Treatment options for early-stage OSCC (stages I and II) typically involve surgical intervention, radiotherapy, and chemoradiation, offering potential for cure [2]. Conversely, patients with advanced OSCC (stages III and IV) face challenges in achieving satisfactory therapeutic outcomes, with a five-year survival rate of less than 50% for those with distant metastases and metastasis continues to be a significant contributor to mortality among patients with OSCC [3, 4]. While a multitude of biomarkers linked to the progression and prognosis of OSCC, including p53, epidermal growth factor receptor (EGFR), and chemokine (C-X-C motif) ligand 9 (CXCL9), have been identified, the availability of molecular targets suitable for clinical intervention remains limited [5, 6]. Consequently, there is considerable clinical merit in investigating the molecular mechanisms underlying OSCC and identifying novel therapeutic targets to enhance patient outcomes.

The emerging body of evidence suggests that transcriptional dysregulation is a significant factor in the development of cancer [7, 8]. The Mediator complex, a highly conserved polyprotein complex consisting of 33 subunits in humans, serves as a key regulator of eukaryotic transcription [9]. The Mediator complex plays a crucial role in various transcriptional processes, including the recruitment of RNA Polymerase II (Pol II), the assembly of the transcription preinitiation complex (PIC), DNA looping, alternative RNA splicing, DNA repair, and transcription termination [10–12]. The Mediator complex has the ability to modulate the transcriptional activity of target genes through various mechanisms. Alterations in its function or constituent subunits can lead to significant implications, potentially contributing to the development of numerous diseases [13, 14], including different types of cancer [15]. Among these Mediator subunits, we are deeply interested in a critical mediator, the Mediator subunit 1 (MED1), which played an important role in cell proliferation, differentiation, metabolism, and internal environmental stability [16, 17]. The preceding investigation conducted by our research team revealed that MED1 exerts regulatory control over the differentiation direction of dental epithelial stem cells at the transcriptional level [18, 19], and participated in the healing of oral wound healing [20]. These results indicated that MED1 has great potential in regulating oral homeostasis and function of the oral mucosal epithelial cell.

Moreover, MED1 is implicated in the pathogenesis of various cancers, with studies demonstrating its overexpression in breast, liver, prostate cancer, and osteosarcoma [21–24], while its downregulation has been observed in colorectal, ovarian cancer, and bladder cancer [25, 26]. The dual nature of MED1 as both an oncogene and tumor suppressor suggest its potential tissue-specific functions. However, the precise role of MED1 in OSCC remains unclear.

The formation of malignant tumors is not only a consequence of tumor cell proliferation, but also a complex interplay of cellular and molecular interactions within the tumor microenvironment, comprised of endothelial cells, fibroblasts, and infiltrating immune cells [27]. Increasing research has indicated that oncogenes play a role in modulating immune system components, indicating a potential mechanism for tumorigenesis [28–30]. As a pivotal regulator of transcription, MED1 is implicated in the intricate processes of tumor development. Its role extends beyond the mere activation or repression of gene expression, potentially influencing the behavior of immune cells within the tumor microenvironment. This complex interaction between MED1 expression in tumor cells and the immune response warrants exhaustive investigation, as it could unveil novel therapeutic targets and strategies for enhancing the efficacy of cancer treatments. Thus, this study aims to explore the expression and functional implications of MED1 in OSCC, as well as its possible molecular mechanisms involving transcriptional regulation and immune response.

## Materials and methods

### Data mining

*Oncomine* database (https://www.oncomine.org) and *TIMER* database (http://timer.cistrome.org) were employed to investigate the MED1 expression in head and neck squamous cell carcinomas (HNSC). The *Gene Expression Omnibus* (*GEO*) database (http://www.ncbi.nlm.nih.gov.geo) was used to assess the expression of MED1 in OSCC. *PrognoScan* database (https://www.prognoscan.org), *Gene Expression Profiling Interactive Analysis* (*GEPIA*) database (https://gepia.cancer-pku.cn) and *UALCAN* database (https://ualcan.path.uab.edu) database were used for survival analysis.

The FPKM (log2[FPKM + 1]) Expression Matrix for the TCGA-HNSC Cohort was downloaded from the *UCSC Xena* Database (https://xenabrowser.net) and patients with anatomic subgroups from the oral cavity (including buccal mucosa, gingiva, floor of the mouth, palate, tongue and lips) were selected for research. A total of 332 oral squamous cell carcinoma samples and 32 control samples were included. Limma was used for the differential analyses of transcriptomic data. The correlation pheatmap of MED1 and Notch signaling pathway-related genes was displayed by the R software package.

### Tissue microarray (TMA) and immunohistochemistry (IHC)

Tissue microarray containing 80 OSCC tissues, 30 para-cancerous tissues, and 10 normal tissues (HN120Oc01) analysis and IHC staining were performed by Xian Zhongke Guanghua Technology Co., Ltd. Tissue sections were incubated with MED1 antibody (1:500, ab243893, Abcam, USA), MMP9 (1:200, A0289, Abclonal, China) antibody and PD-L1 antibody (1:200, A1645, Abclonal, China). Two independent pathologists manually scored the TMA core intensity. The expression levels were assessed based on the staining intensity (0 for no staining, 1 for weak staining, 2 for moderate staining, and 3 for strong staining) and the positive cell ratio (1 for < 25%, 2 for 26 to < 50%, 3 for 51 to < 75%, and 4 for > 75% cell). The final score was obtained by summing the percentage of positive cells and the staining intensity score.

### Cell culture and MED1 knockdown/ overexpression OSCC cell lines construction

The human oral keratinocyte (HOK) was purchased from Tongpai Biotechnology Co., Ltd, (Shanghai, China). Human OSCC cell lines Cal-27, UPCI-SCC-090, SCC-9, UPCI-SCC-154 and mouse OSCC cell line SCC-7 were purchased from the American Type Culture Collection (ATCC, USA). The cell lines were validated by STR analysis. HOK was maintained in cultured in defined keratinocyte serum-free medium (Defined keratinocyte-SFM, Gibco, USA). Cal-27 and UPCI-SCC-154 were cultured in DMEM (Hyclone, USA) supplemented with 10% FBS (HAKATA, China) and 1% penicillin/streptomycin (Solarbio, China) at 37 °C in a humidi-fied 5% CO_2_ atmosphere. While UPCI-SCC-090 cells were kept in high glucose DMEM medium (Hyclone, USA), SCC-9 cells were kept in DMEM/F12 medium (Hyclone, USA), and SCC-7 cells were kept in 1640 medium (Yuanpei, China). The MED1 shRNA/overexpressed lentiviral vectors were constructed by Jikai (Shanghai, China). Transfection was performed in accordance with the instructions provided by the manufacturer.

### RNA extraction and quantitative real-time polymerase chain reaction (qRT-PCR) analysis

Total RNA was extracted by Trizol reagent (ET101-01-V2, TransGene, China) and cDNA was synthesized using the TransScript All-in-One First-Strand cDNA synthesis Supermix (AT341, TransGen, China). qRT-PCR was performed using the PerfectStart Green qPCR SuperMix (AQ602, TransGen, China). The glyceraldehyde-3-phosphate dehydrogenase (GAPDH) gene was used as control. The primers used can be found in Table S1 of Supplementary Material 1. The relative gene expression levels were calculated based on the 2^−ΔΔCt^ method.

### Western blot (WB) analysis

Western blot (WB) analysis was performed as previously described [20], Anti-GAPDH, anti-MED1, anti-MMP9, anti-PD-L1, anti-NICD, anti-HES1 antibodies and HRP-conjugated secondary antibodies were used. A chemiluminescence system (ECL Kit) from Servicebio (G2074, China) was used to visualize the immunoreaction and quantified in ChemiDoc XRS Imaging System. The antibody details are shown in Table S2 of Supplementary Material 1.

### Cell counting kit 8 (CCK8) experiment

CCK8 assay was used to evaluate cell proliferation. A total of 1500 cells per well were inoculated into 96-well plates and cultured for 24 h. And then, 10 µL CCK8 solution (CA1210, Solarbio, China) was added to each well. The cells were incubated at 37 °C for 2 h and the OD value was measured by an enzyme labeling instrument at 450 nm wavelength.

### Immunocytochemistry (ICC) staining

ICC assays were performed to evaluate Ki67 expression in MED1 knockdown and MED1 overexpression OSCC lines as well as negative control cell lines. Briefly, cells were performed with 4% paraformaldehyde fixation, permeabilization with 0.2% Triton X-100 solution and covered with blocking solution (5% BSA in PBS) at RT (room temperature) for 1 h, followed by overnight incubation with primary antibodies: anti-Ki67 (1:500, ab92742, Abcam, USA). After washing with PBST, cells were incubated with secondary antibodies: goat anti-rabbit Alexa Fluor 488 (1:1000, RS3211, Immunoway, USA), and sealed with a fluorescence quenching sealing tablet containing DAPI (C1005, Beyotime, China).

### Plate colony formation assay

Cell colony formation ability was assessed using the plate colony formation assay. Specifically, 800 cells were seeded into each well of a 6-well plate and incubated for about two weeks until visible colonies formed. Subsequently, cells were fixed with 4% paraformaldehyde for 10 min and stained with 0.1% crystal violet solution (G1063, Solarbio, China) for 20 min. The Leica stereomicroscope was used to count the number of wells with clone spheres ($>$ 50 cells), and the colony formation rate was calculated using the formula: (number of clone spheres/number of successfully inoculated) $\times$ 100%.

### Transwell invasion assay

The cell invasion assay was performed using Transwell inserts containing an 8.0 μm pore size membrane (Corning, USA). 2 × 10^4^ cells suspended in 200 μl medium were seeded into the upper chamber pre-coated with Matrigel Matrix (356,231, BD Biosciences, USA), and 500 μl medium containing 30% FBS was added to the lower chamber. After incubation for 24 h, cells that did not invaded through the membrane were wiped with a cotton swab. Next, fixed the cells on the bottom surface of the membrane with 4% paraformaldehyde for 10 min, and stained them with 0.1% crystal violet solution. The invading cells were imaged using an inverted microscope (Zeiss, GER).

#### Cell scratch assay

Cell migration was assessed using the scratch assay. Briefly, cells were cultured as monolayers in 6-well plates. Scratches were made using a 200 μl pipette tip and the wells were washed with PBS to remove floating cells. Migrated areas were photographed every 6 h and were later analyzed using Image J software. Cell migration rate was calculated as the following formula: (original scratch area $-$ the point scratch area)/original scratch area $\times$ 100%.

#### Lung metastasis experiment

MED1-knockdown SCC-7 cells, MED1-overexpression SCC-7 cells and their vector control cells were used to generate the animal model. C57BL/6 J mice were ordered from Sipeifu Biotechnology Co., Ltd. A lung metastasis model was established using tail vein injections. 1 × 10^7^ of the indicated cells were suspended in 1 mL of PBS and injected into the lateral tail vein of 8-week-old female C57BL/6 J mice (10-12 mice per group). The health and weight of the mice were monitored every 4 days. At 20 days after injection, all mice were subjected to live imaging by the small animal living imaging system (PerkinElmer, USA) and then sacrificed. The lung, spleen, livers, heart, and kidney were harvested. A count of metastatic foci was performed on the surface of lungs.

#### Hematoxylin and eosin (HE) staining

Pathological changes of each organ of two group mice were observed by HE staining. Briefly, the organs were fixed in 4% paraformaldehyde, embedded in paraffin, and longitudinally sectioned. After deparaffinization and rehydration, the tissue sections were stained with hematoxylin solution (G1140, Solarbio, China) for 5 min, then the sections were stained with eosin solution (G1100, Solarbio, China) for 2 min and followed by dehydration with gradually increasing concentration of alcohol and clearing in xylene. The images of HE staining were photographed with a microscope (Nikon, Japan).

#### MMP9 promoter dual-luciferase reporter assay

MMP9 promoter regulation was investigated using dual-luciferase reporter assay. MED1-knockdown SCC-9 and UPCI-SCC-154 cell lines were seeded in 24-well culture plates, and transfected with psiCHECK-2-MMP9 plasmid which containing MMP9 promoter fragment (-650 ~  + 19 bp). After transfection for 48 h, luciferase activities were measured using the dual-luciferase reporter assay system (E1910, Promega, USA) according to the manufacturer’s protocol. Firefly luciferase activity was normalized to Renilla luciferase activity and presented as relative luciferase activity. MMP9 reporter dual-luciferase vector were constructed by Uptbio Co., Ltd. (Hunan, China).

#### Rescue experiments

Rescue experiments contained two independent experiments, the cell scratch assay and Transwell assay. The SCC-7-ShMED1 cells were cultured with or without 10 ng/mL MMP9 recombinant protein (RP00103, Abclonal, China) for 24 h. Cells were collected for scratch assay and Transwell assay following enzymatic digestion with 0.05% trypsin. The formula for calculating cell recovery efficiency is as follows:

$$\frac{ShMED1+MMP9\;group\;cell\;migration\;rate-\;ShMED1\;group\;cell\;migration\;rate}{ShMED1\;group\;cell\;migration\;rate}\times100\%;$$

$$\frac{\mathrm{ShMED}1+\mathrm{MMP}9\;\mathrm{group}\;\mathrm{invaded}\;\mathrm{cell}\;\mathrm{numbers}-\;\mathrm{ShMED}1\;\mathrm{group}\;\mathrm{invaded}\;\mathrm{cell}\;\mathrm{numbers}}{\mathrm{ShMED}1\;\mathrm{group}\;\mathrm{invaded}\;\mathrm{cell}\;\mathrm{numbers}}\times100\%.$$

#### Chromatin immunoprecipitation (ChIP) assay

ChIP was performed as follows. MED1-knockdown OSCC cell lines were seeded in 10-cm culture plates and 1 × 10^7^ cells were harvested. To generate the cross-link, cells were processed with 1% formaldehyde and maintained at RT for 10 min. Subsequently, glycine was then added to terminate the cross-link reaction. Cells were gathered in lysis buffer after washing three times with pre-cooled PBS. Next, the lysates were incubated with Protein A/G (RM02915, Abclonal, China), which was incubated at least 3 h with specific antibody at 4 °C before incubation. After washing three times with elution buffer, the DNA–protein-antibody complex was incubated overnight at 65 °C to de-cross-link. DNA was purified using DNA purification columns (RK30100, Abclonal, China) for subsequent qRT-PCR analysis. Primer sequences were as follows:

$$\mathrm{ChIP}-\mathrm{MMP}9\ \mathrm{forward}:\mathrm{GGAGGTGGTGTAAGCCCTTT},$$

$$\mathrm{ChIP}-\;\mathrm{MMP}9\;\mathrm{reverse}:\;\mathrm{AGGGCAGAGGTGTCTGACTG}.$$

#### Sort and purify CD8^+^ T cells in human peripheral blood

Human peripheral blood was obtained from healthy volunteers. Peripheral blood mononuclear cells (PBMCs) were isolated by Ficoll separating solution (17,544,602, GE, USA) through density gradient centrifugation. CD8^+^ T cells were isolated from human PBMCs using the CD8^+^ T Cell Isolation Kit (130–096-495, Miltenyi, GER), an LS Column (130–042-401, Miltenyi, GER), and a MidiMACS™ Separator (130–042-501, Miltenyi, GER). The isolated CD8^+^ T cells were collected into new EP tubes.

#### Flow cytometric analysis

The harvested CD8^+^ T cells were washed with PBS and incubated with PE anti-CD8 antibody (344,705, Biolegend, USA) for 4 min at 4 °C. After washing three times with PBS, the cells were resuspended in PBS and analyzed by flow cytometry.

For the mouse spleen samples, the tissues were ground with a plastic rod on ice and filtered through a 70 μm nylon strainer. Cells were incubated in 2 mL red blood cell lysis buffer (R1010, Solarbio, China) for 5 min at RT. Then the cells were suspended in 2% BSA/PBS buffer and costained with LIVE/DEAD fixable dead cell stain (L34969, Invitrogen, USA) as well as the following antibodies: anti-CD45 (APC conjugated, 368,516, Biolegend, USA); anti-CD3 (PerCP conjugated, 100,218, Biolegend, USA); anti-CD4 (FITC conjugated, 100,510, Biolegend, USA); anti-CD8 (PE conjugated, 100,722, Biolegend, USA). After incubation with antibody for 1 h, the cells were washed with PBS and analyzed by flow cytometry.

Cells were sorted on a FACSVerse (Becton–Dickinson, USA) and samples were run on a LSRFortessa (Becton–Dickinson, USA). All data were analyzed with FlowJo software.

#### Human CD8^+^ T cell activation and cocultured with tumor cells

The CD8^+^ T cells were activated and expended by the addition of recombinant human IL-2 protein (GMP-TL777, T&L Biotechnology, China) and anti-CD3/CD28 beads (ACROBiosystems, China) for 72 h. Coculture assays were performed in a 96-well plate format in which CD8^+^ T cells were cocultured with OSCC cells at a 5:1 effector to target [E: T] ratio. The Plates were centrifuged at 400 g for 5 min to ensure cell-to-cell contact and the co-cultures were cultured in a 5% CO_2_ incubator at 37 °C for 24 h. The culture supernatant was collected and CCK8 assay was performed to assess tumor cell viability after CD8^+^ T cell coculture.

#### Enzyme-linked immunosorbent assay (ELISA)

We used the ELISA kit to detect the supernatant interferon gamma (IFN-γ, KHC4022, Thermo Fisher, USA) and IL-2 (Interleukin-2, RK00002, Abclonal, China) content. Briefly, cell lysate was added and incubated for 2 h at 37 °C. The human IFN-γ/ IL-2 conjugate was added to each well after washing for three times with wash buffer and then incubated for 1 h at 37 °C. The substrate solution was added to each well subsequently and incubated for 30 min at 37 °C. Last, the reaction was ended by stop solution. In under 30 min, OD of each sample was detected and corrected at 450 nm and 540 nm respectively. IFN-γ/ IL-2 contents were calculated from OD according to protein standards.

#### Statistical analyses

Data analyses were performed using GraphPad Prism 9.0 (GraphPad Software, La Jolla, USA) with unpaired or paired analyses as indicated. For experiments where more than two groups are compared, statistical analyses were performed using one-way ANOVA followed by two-tail Student t-tests. Statistical annotations were denoted with asterisks as follows: **** *P* < 0.0001, *** *P* < 0.001, ** *P* < 0.01, * *P* < 0.05, and not significant (ns) *P* > 0.05.

## Results

### MED1 is upregulated in OSCC patients and correlated with shorter overall survival

**Fig. 1:**
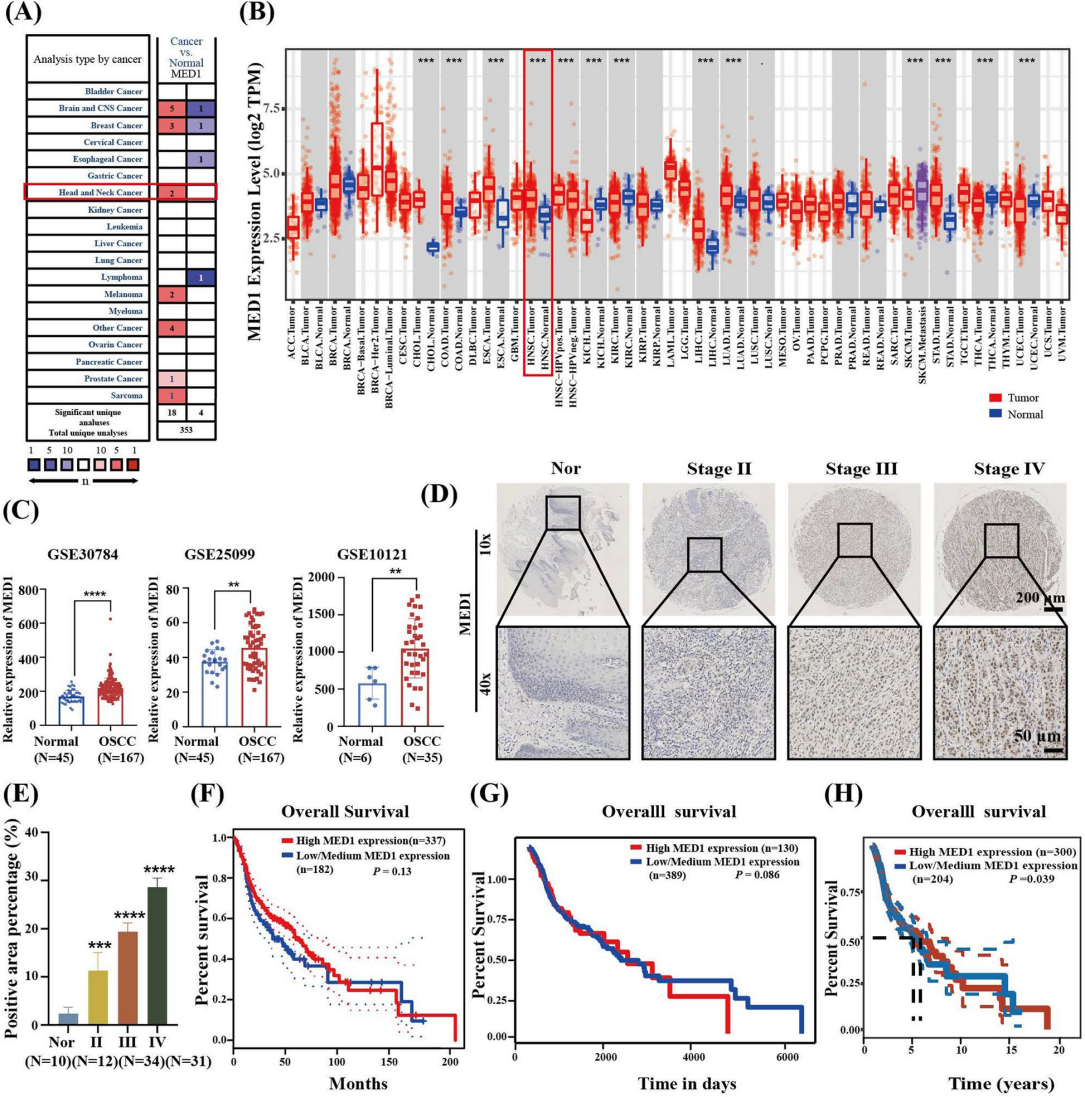
MED1 is upregulated in OSCC patients and associated with poor prognosis. **A** Analysis of the mRNA levels of MED1 (cancer vs. normal) in multiple solid cancers from Oncomine Database. **B** Analysis of the MED1 expression level (tumor vs. normal) in multiple solid cancers from TIMER Database. **C** mRNA expression of MED1 in 3 samples (GSE30784, GSE25099, and GSE10121) from the GEO database. **D** Immunohistochemical staining of MED1 in OSCC tissue microarray (TMA). **E** Quantification of immunostaining results. **F** Kaplan–Meier analysis of overall survival of OSCC patients from GEPIA database stratified by MED1 levels. **G** Kaplan–Meier analysis of overall survival of OSCC patients from UALCAN database stratified by MED1 levels. **H** Kaplan–Meier analysis of overall survival of OSCC patients from TCGA database stratified by MED1 levels. Scale bar = 200 µm (10 ×), and 50 µm (40 ×), Bars = means ± SD. ** *P* < 0.01, ***P < 0.001, ****P < 0.0001. OSCC, oral squamous cell carcinoma

Initially, we utilized the Oncomine database to conduct a comparative analysis of MED1 transcription levels in diverse tumor tissues and their corresponding normal tissues. The findings revealed a notable upregulation of MED1 expression in individuals diagnosed with head and neck squamous cell carcinomas (HNSC) (Fig. 1A), a result further corroborated by data from the TIMER databases (Fig. 1B). HNSC originates from various sites in the head and neck area, including the larynx, oropharynx, hypopharynx and oral cavity. In order to mitigate the impact of intra-tumoral heterogeneity on the outcomes, a diverse subgroup of OSCC was selected for subsequent validation using the GEO database. Analysis of the randomly selected datasets GSE30784, GSE25099, and GSE10121 revealed that the expression levels of MED1 were elevated in OSCC tissues compared to normal samples (Fig. 1C). TMA analysis revealed a significant upregulation of MED1 expression in OSCC tumors compared to normal tissues across all stages of disease progression, with a trend towards higher expression levels in advanced stages (Fig. 1D and E). Furthermore, our study demonstrated a modest correlation between increased MED1 expression and decreased overall survival in OSCC patients, as evidenced by Kaplan–Meier analysis using three distinct databases: GEPIA database (Fig. 1F), UALCAN database (Fig. 1G), and TCGA database (Fig. 1H). Collectively, these findings indicate that MED1 is overexpressed in OSCC, and has a modest correlation with the overall survival.

### MED1 is highly expressed in human OSCC cell lines and has no significant effect on cell proliferation

**Fig. 2:**
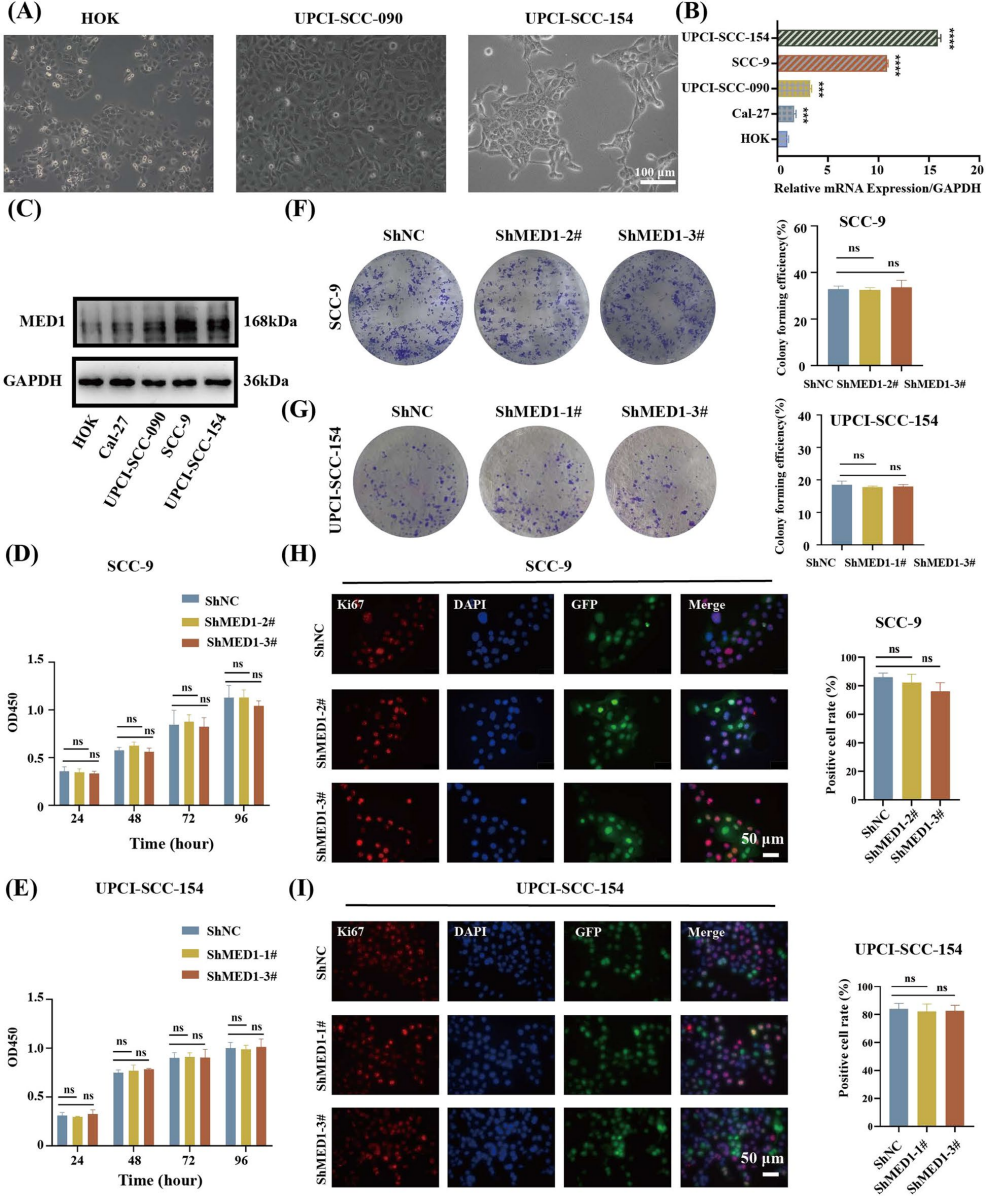
MED1 is highly expressed in human OSCC cell line and has no significant effect on metastatic OSCC cells proliferation. **A** Cell morphology of human oral keratinocytes (HOK), nonmetastatic oral squamous cell carcinoma UPCI-SCC-090, and metastatic oral squamous cell carcinoma UPCI-SCC-154. **B** mRNA levels of MED1 in HOK and four OSCC lines tested by qRT-PCR. *n* = 3 independent experiments. **C** Protein levels of MED1 in HOK and four OSCC lines tested by WB. **D** CCK8 to detect SCC-9 cell proliferation change after MED1 knockdown. *n* = 3 independent experiments. **E** CCK8 to detect UPCI-SCC-154 cell proliferation change after MED1 knockdown. *n* = 3 independent experiments. **F** Plate colony formation assay to detection of clonality in SCC-9 cells following MED1 knockdown. *n* = 3 independent experiments. **G** Plate colony formation assay to detection of clonality in UPCI-SCC-154 cells following MED1 knockdown. *n* = 3 independent experiments. **H** Immunofluorescence staining and quantitative analysis of proliferation marker Ki67 after MED1 knockdown in SCC-9 cells. *n* = 3 independent experiments. **I** Immunofluorescence staining and quantitative analysis of proliferation marker Ki67 after MED1 knockdown in UPCI-SCC-154 cells. *n* = 3 independent experiments. Scale bar = 100 µm (10 ×), and 50 µm (40 ×). Bars = means ± SD. ns means nonsignificant, *** *P* < 0.001, **** *P* < 0.0001

Subsequently, the in vitro experiments were conducted to assess the impact of MED1 on the biological behaviors of human OSCC cells. Four human OSCC cell lines were selected for this study: Cal-27, UPCI-SCC-090, SCC-9, and UPCI-SCC-154. Cal-27 and UPCI-SCC-090 cells are classified as nonmetastatic OSCC cell lines, while SCC-9 and UPCI-SCC-154 are categorized as metastatic OSCC cell lines [31, 32]. It was observed that UPCI-SCC-090 cells exhibited an epithelial-like appearance similar to human oral keratinocytes (HOK), whereas UPCI-SCC-154 cells displayed a fibroblast-like morphology with a spindle-shaped structure, which was the typical aggressive cells morphology (Fig. 2A). The MED1 mRNA levels of five cell lines were analyzed using qRT-PCR. Results indicated higher mRNA expression of MED1 in SCC-9 and UPCI-SCC-154 cells compared to Cal-27 and UPCI-SCC-090 cells. Furthermore, all four OSCC cell lines exhibited elevated MED1 mRNA expression compared to HOK cells (Fig. 2B). The protein expression levels were in agreement with the mRNA results (Fig. 2C).

Given the importance of invasion and metastasis mechanisms in cancer therapy, two metastatic OSCC cell lines were chosen for further experimentation. We constructed MED1 stable knockdown and overexpression cells in SCC-9 and UPCI-SCC-154 cell lines. The tumor cells were transfected with negative control ShRNA (ShNC), ShMED1-1#, ShMED1-2#, and ShMED1-3#. Green fluorescent protein (GFP) was used as the transfection marker (Fig. S1A and S1C). The expression of the MED1 gene was significantly reduced, as confirmed by qRT-PCR. SCC-9-ShMED1-2#, SCC-9-ShMED1-3#, UPCI-SCC-154–1#, and UPCI-SCC-154–3#, with higher silencing efficiency, were selected for further protein level analysis. Western blot (WB) demonstrated that MED1 protein expression was also significantly downregulated in these cells (Fig. S1B and S1D). SCC-9 and UPCI-SCC-154 cells were further transfected with negative control lentivirus and MED1-overexpressing lentivirus (Fig. S1E and S1G). Overexpression of MED1 were verified using qRT-PCR and WB (Fig. S1F and S1H).

To verify the effect of MED1 on cell proliferation, we firstly performed a CCK-8 experiment in the SCC-9 and UPCI-SCC-154 cells. As shown in Fig. 2D and E, the cell proliferation was not significantly changed after MED1 knockdown. In addition, the plate cloning assay also confirmed the results (Fig. 2F and G). Moreover, Ki67 immunofluorescence staining was utilized for further verification. The percentage of Ki67 positive cells on the control groups were not statistically different from that on MED1 knockdown groups (Fig. 2H and I). In order to verify the role of MED1 in metastatic OSCC cells, we also performed the three experiments in SCC-9 and UPCI-SCC-154 cells that were overexpressing MED1. In line with the findings from the knockdown experiments, the overexpression of MED1 did not alter the proliferation capacity of SCC-9 and UPCI-SCC-154 cells (Fig. S2). Collectively, these results indicate that MED1 does not influence the proliferation of metastatic OSCC cells in vitro.

### MED1 promotes metastatic OSCC cells migration and invasion in vitro and in vivo

**Fig. 3:**
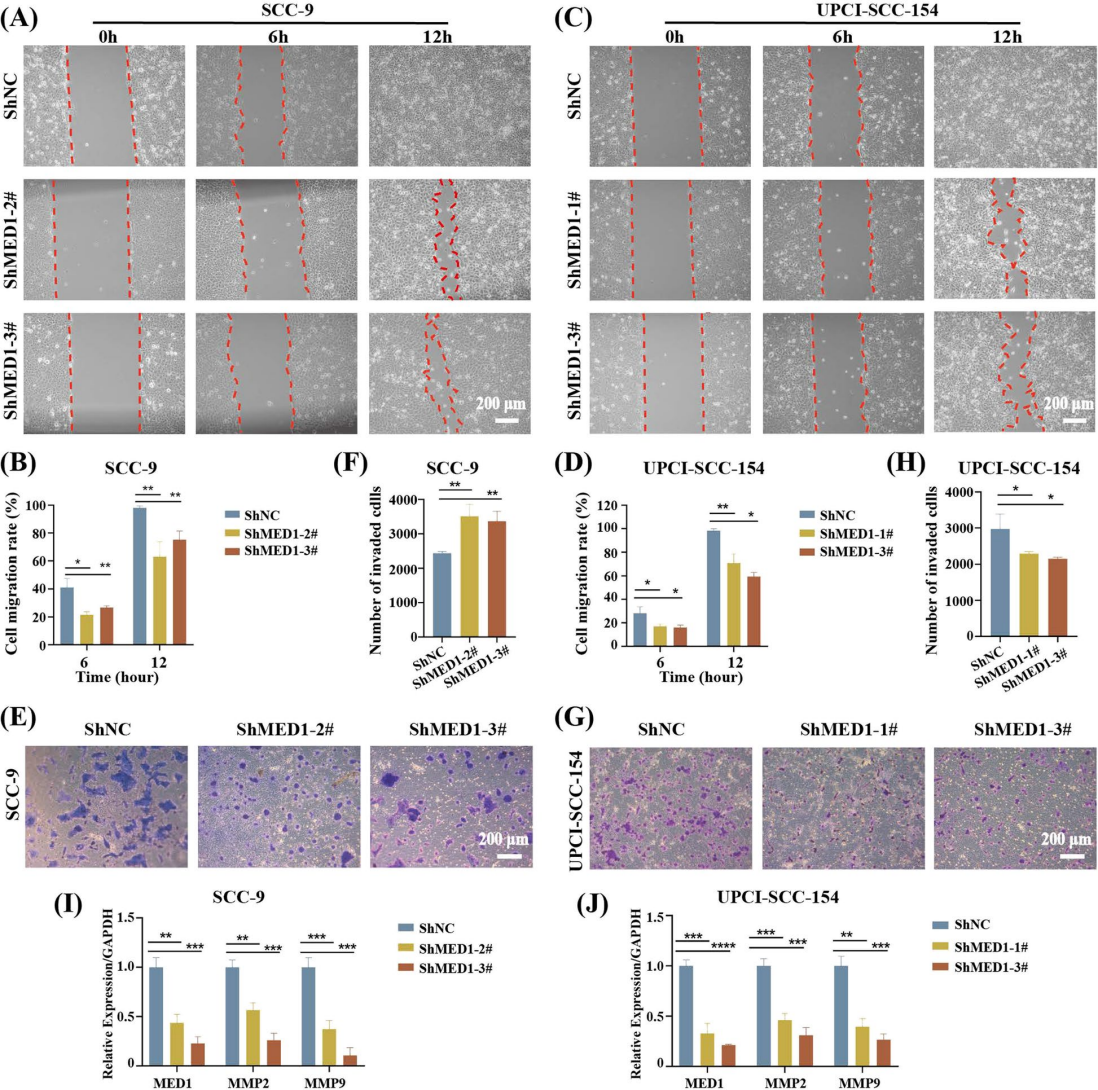
MED1 knockdown inhibits metastatic OSCC cells migration and invasion in vitro. **A** Cell scratch assay to examine SCC-9 cells migration ability after MED1 knockdown. **B** Quantitative analysis of the cell migration ratio in (**A**). *n* = 3 independent experiments. **C** Cell scratch assay to examine UPCI-SCC-154 cells migration ability after MED1 knockdown. **D** Quantitative analysis of the cell migration ratio in (**C**). *n* = 3 independent experiments. **E** Transwell invasion assay to examine SCC-9 cells invasion ability after MED1 knockdown. **F** Quantitative analysis of the number of invasive cells in (E). *n* = 3 independent experiments. **G** Transwell invasion assay to examine UPCI-SCC-154 cells invasion ability after MED1 knockdown. **H** Quantitative analysis of the number of invasive cells in (**G**). *n* = 3 independent experiments. **I** MMP2 and MMP9 gene expression assessed by qRT-PCR in SCC-9 cells after MED1 knockdown. *n* = 3 independent experiments. **J** MMP2 and MMP9 gene expression assessed by qRT-PCR in UPCI-SCC-154 cells after MED1 knockdown. *n* = 3 independent experiments.Scale bar = 200 µm (10 ×). Bars = means ± SD.* *P* < 0.05, ** *P* < 0.01, *** *P* < 0.001, **** *P* < 0.0001

We then assessed the metastatic potential of MED1. Cell scratch assay and transwell invasion assay indicated that the knockdown of MED1 significantly inhibited cell migration and invasion in SCC-9 and UPCI-SCC-154 cells (Fig. 3A-H). Conversely, migration and invasion were increased in SCC-9 and UPCI-SCC-154 cells overexpressing MED1 (Fig. S3A-3H). It is important to note that matrix metalloproteinases (MMPs) play a critical role in remodeling the basement membrane and facilitating the cell invasion process [33]. MMP2 and MMP9 play a crucial role in tumor cell invasion and metastasis by degrading the basal membrane [34–36]. In this study, we investigated the expression of MMP2 and MMP9 as potential markers to validate MED1-induced migration and invasion. Our qRT-PCR analysis demonstrated that MMP2 and MMP9 expression levels were decreased upon MED1 knockdown (Fig. 3I and J), and increased following MED1 overexpression (Fig. S3I and S3J). Furthermore, following knockdown or overexpression of MED1, the change in MMP9 expression was found to be more pronounced compared to that of MMP2, indicating a greater impact of MED1 on MMP9. The results of bioinformatics analysis also indicate that the expression levels of MED1 exhibit a positive correlation with both MMP2 and MMP9. Notably, the correlation between MED1 and MMP9 is especially pronounced (Fig. S4).

**Fig. 4:**
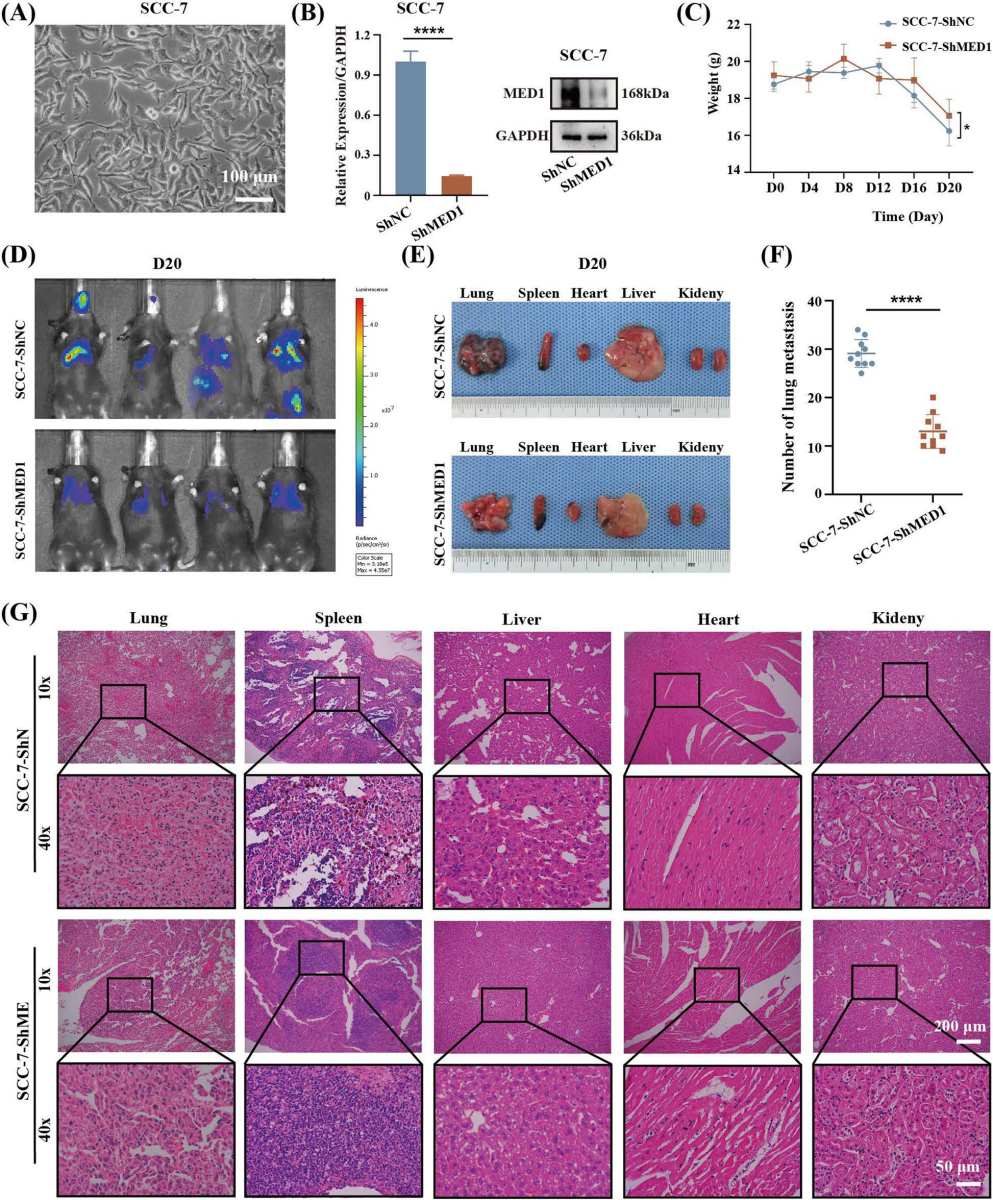
MED1 knockdown inhibits tumor metastasis in vivo. **A** Cells were observed under a light microscope. **B** Knockdown efficiency of MED1 in SCC-7 cells via qRT-PCR and WB. *n* = 3 independent experiments. **C** Body weight changes of mice following tumor injection. *n* = 10–12 mice per group. **D** Tumors were visualized by luciferase living imaging. **E** Gross view of the lung, spleen, heart, liver, and kidney. **F** Number of lung metastatic nodules of the mice. *n* = 10 mice per group. **G** HE staining images of various organs. Scale bar = 200 µm (10 ×), and 50 µm (40 ×). Bars = means ± SD. * *P* < 0.05, **** *P* < 0.0001

Subsequently, in order to assess the role of MED1 in metastatic potential in vivo, SCC-7 (a murine OSCC line [37]) cells in the logarithmic growth phase (Fig. 4A) were used for the assay. The cells were stably transfected with negative control ShRNA (ShNC) and MED1-knockdown ShRNA (ShMED1) and the knockdown efficiency was verified by qRT-PCR and WB (Fig. 4B). The results from the cell scratch assay and transwell invasion assay demonstrated that the knockdown of MED1 markedly suppressed cell migration and invasion in SCC7 cells (Fig. S6A and S6C). Then these cells were injected into C57BL/6 J mice via the tail vein for monitoring. As shown (Fig. 4C), the weight loss in ShMED1 group was significantly lower than that in ShNC group at day 20, indicating that the cancer development in ShMED1 group was relatively slow. Luciferase assays revealed a substantial decrease in lung and other organ metastases in the ShMED1 group of mice compared to the control group (Fig. 4D). Macroscopic examination corroborated these findings, demonstrating a notably lower incidence of lung metastasis in the ShMED1 group relative to the control group, while no significant differences were observed in metastases to other organs (Fig. 4E). Quantitative analysis confirmed that the number of lung metastases in the ShMED1 group was significantly reduced compared to the control group (Fig. 4F). The changes in lung tissue and other organs histopathology were observed using Hematoxylin–eosin (HE) staining. It is well-known that the degree of cell differentiation was inversely correlated with the degree of malignancy, specifically, a lower degree of differentiation was associated with a higher degree of malignancy. Within the ShMED1 group, well-differentiated lung squamous cell carcinoma was identified, characterized by intercellular bridges and keratinization within the cancer nest. Conversely, the control group exhibited lung tumors with prominent atypia and low differentiation. Furthermore, tumors in the liver and spleen of the ShMED1 group displayed better differentiation compared to those in the control group. There was no significant difference in the degree of tumor differentiation between the two groups in the kidneys. No metastases were observed in the hearts of mice in both groups (Fig. 4G). Cardiac metastases occur infrequently may due to the faster velocity of blood flow in the heart. In contrast, overexpression of MED1 in SCC‐7 showed significantly enhanced invasion ability in vitro and in vivo (Fig. S5, Fig. S6B and S6D).

Taken together, these findings indicate that MED1 may facilitate the migration and invasion of cancer cells in vitro, as well as metastasis in vivo.

### MED1 regulates metastatic OSCC cells migration and invasion through the modulation of MMP9

**Fig. 5:**
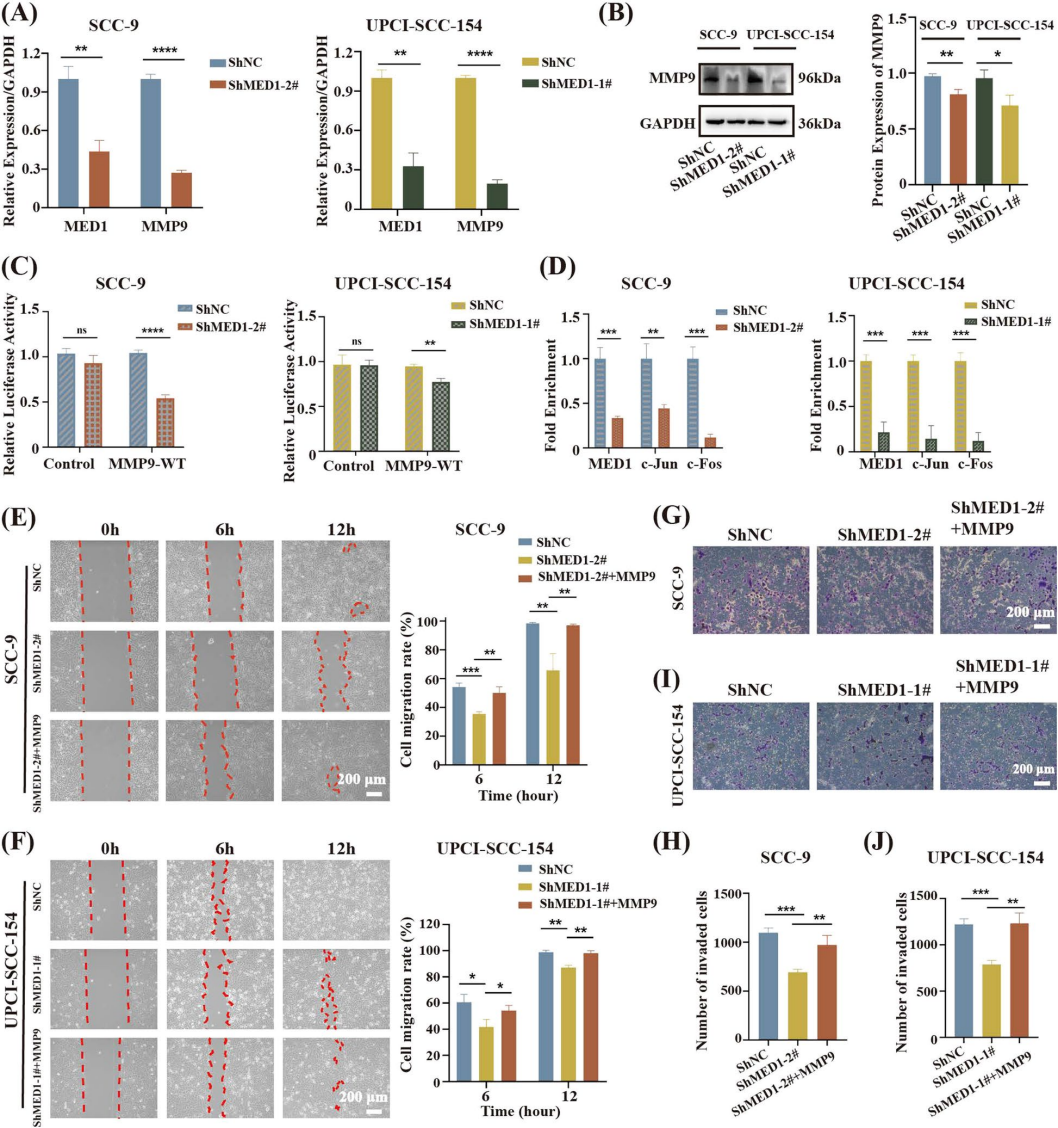
MED1 regulates metastatic OSCC cells migration and invasion through the modulation of MMP9. **A** MMP9 gene expression assessed by qRT-PCR. *n* = 3 independent experiments. **B** MMP9 protein expression assessed by WB. *n* = 3 independent experiments. **C** The luciferase activity of MMP9 promoter detected by luciferase reporter assay. *n* = 3 independent experiments. **D** The recruitments of cofactors AP-1(c-Jun/c-Fos) on MMP9 promoter in SCC-9 cells and UPCI-SCC-154 cells by CHIP assay and qRT-PCR. *n* = 3 independent experiments. **E** Cell scratch assay to determine the changes in migration abilities of SCC-9 cells after adding exogenous MMP9 and quantitative analysis. *n* = 3 independent experiments. **F** Cell scratch assay to determine the changes in migration abilities of UPCI-SCC-154 cells after adding exogenous MMP9 and quantitative analysis. *n* = 3 independent experiments. **G** Transwell assay to determine the changes in invasion abilities of SCC-9 cells after adding exogenous MMP9 and quantitative analysis (**H**). *n* = 3 independent experiments. **I** Transwell assay to determine the changes in invasion abilities of UPCI-SCC-154 cells after adding exogenous MMP9 and quantitative analysis (**J**). *n* = 3 independent experiments. Scale bar = 200 µm (10 ×). Bars = means ± SD.* *P* < 0.05, ** *P* < 0.01, *** *P* < 0.001, **** *P* < 0.0001

In order to investigate the regulatory role of MED1 in the migration and invasion of metastatic OSCC, our study examined the impact of MED1 knockdown on MMP9 levels. qRT-PCR analysis revealed a decrease in MMP9 mRNA levels in SCC-9 and UPCI-SCC-154 cells following MED1 knockdown (Fig. 5A). WB analysis confirmed a corresponding decrease in MMP9 protein expression upon downregulation of MED1 (Fig. 5B). To further investigate the relationship between MED1, MMP9, and the clinical implications in OSCC, we examined the protein expression of MED1 and MMP9 in the OSCC tissue microarray using IHC staining. The protein levels of MMP9 exhibited a significant positive correlation with those of MED1 (Fig. S7A and S7B). These data suggest that MED1 may be a key regulator of MMP9 gene expression. To further validate the regulatory role of MED1 on MMP9 activity, we transfected SCC-9 and UPCI-SCC-154 cells with MMP9 promoter-driven (-650 ~  + 19 bp wild type, hMMP9p-Luc, WT) luciferase reporter genes simultaneously with MED1 ShRNA expression vector. The results of luciferase reporter assay showed that MED1 knockdown repressed the luciferase activity of the MMP9 promoter (Fig. 5C), suggesting that MED1 may modulate MMP9 transcription by regulating its promoter. We employed a series of human MMP9 promoter luciferase reporter gene vectors, including the wild-type (wt, -670 ~  + 19), fragment deletion mutants (ΔA, ΔB, ΔC, ΔD, ΔE), and point mutants (mNF-κB, mAP-1) to identify and analyze the specific response region of MED1 on the MMP9 promoter (The schematic diagram is shown in Fig. S8A). The findings indicated that, similar to the wild-type vector, the luciferase activities of the fragment deletion mutants (ΔA, ΔB, ΔC, ΔD, ΔE) and the mNF-κB point mutant promoter vectors were significantly reduced following MED1 knockdown. However, the activity of the AP-1 site point mutant promoter (mAP-1) remained unaffected by MED1 expression (Fig. S8B and S8C). This observation implies that MED1 modulates MMP9 transcription by binding to the proximal AP-1 site on the MMP9 promoter. To explore the impact of MED1 on endogenous MMP9 promoters, subsequent ChIP experiments were conducted. The results indicated that the relative recruitment levels of MED1 at the AP-1 site were comparable to those of the AP-1 proteins, c-Jun and c-Fos (Fig. S8D). Furthermore, sequential ChIP experiments demonstrated that MED1 can co-bind with c-Jun and c-Fos at the AP-1 site proximal to the MMP9 promoter (Fig. S8E). This finding suggests that MED1 regulates MMP9 transcription by participating in the formation of the AP-1 complex. Further experimental results showed that a significant reduction in the recruitment of c-Jun and c-Fos to the MMP9 promoter following MED1 silencing, as illustrated in Fig. 5D. These results provide further evidence supporting the role of MED1 in modulating the recruitment of AP-1(a heterodimer composed of c-Jun and c-Fos that induces the production of MMPs [38]) proteins to the endogenous MMP9 promoter and the formation of the AP-1 transcription complex, thereby influencing the transcriptional regulation of MMP9.

In order to further investigate the role of MED1 in regulating the migration and invasion of metastatic OSCC through modulation of MMP9 expression, a rescue experiment was conducted. Results from cell scratch assays demonstrated that the increased expression of MMP9 recovered the migration capacity of MED1 knockdown cancer cells (Fig. 5E and F). Additionally, Transwell invasion assays revealed that the invasion ability of SCC-9-ShMED1 and UPCI-SCC-154-ShMED1 cells was restored upon reintroduction of MMP9 (Fig. 5G to J). It was calculated that the cell migration recovery rate after adding MMP9 recombinant protein was 30.427% ± 0.015 (SCC-9 cell), and 24.207% ± 0.012 (UPCI-SCC-154 cell) at 12 h. Cell invaded recovery rate after adding MMP9 recombinant protein was 39.939% ± 0.064 (SCC-9 cell), and 55.876% ± 0.047 (UPCI-SCC-154 cell). These findings suggest that MMP9 acts as a downstream target of MED1 in the context of OSCC progression.

In summary, our result demonstrates that MED1 knockdown significantly inhibits migration and invasion in metastatic OSCC cells by downregulating MMP9 expression.

### MED1 regulates PD-L1 expression through Notch signaling pathway and influences CD8^+^ T cell function

**Fig. 6:**
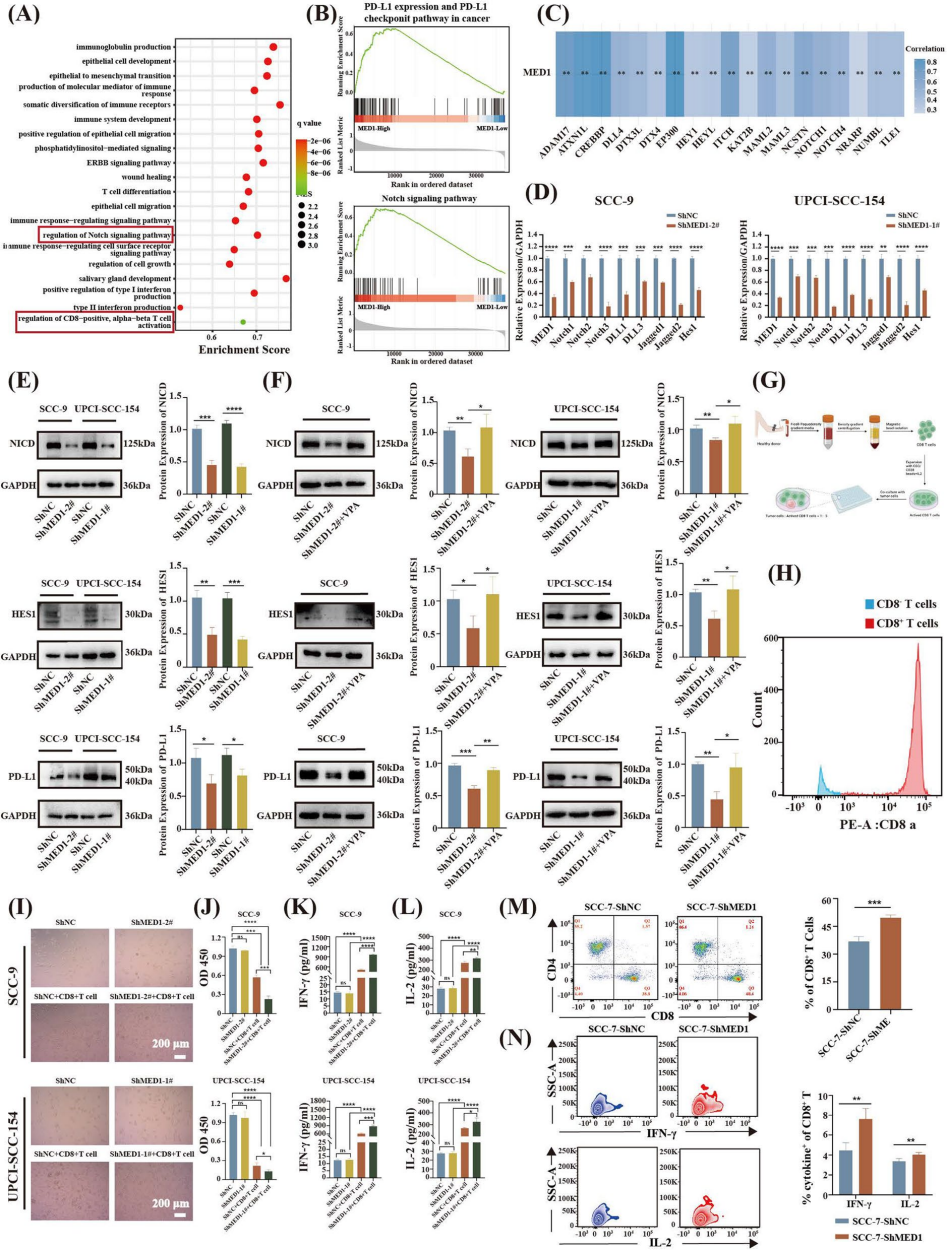
MED1 regulates PD-L1 expression and CD8^+^ T cell function through Notch signaling pathway. **A** GO analysis showing biological processes of differential gene enrichment. **B** Gene set enrichment analysis (GSEA) of differentially expressed genes. **C** Heat map of the correlation of MED1 gene with Notch signaling pathway. **D** Expression analysis of Notch signaling pathway-related genes in response to MED1 knockdown by qRT-PCR in SCC-9 and UPCI-SCC-154 cells. *n* = 3 independent experiments. **E** Expression analysis of NICD, HES1, and PD-L1 in response to MED1 knockdown by WB in SCC-9 and UPCI-SCC-154 cells. *n* = 3 independent experiments. **F** NICD, HES1, and PD-L protein expression by WB with the addition of Notch agonist VPA in SCC-9 and UPCI-SCC-154 cells. *n* = 3 independent experiments. **G** The schematic diagram of CD8^+^ T cells extraction and functional verification. **H** Flow cytometric analysis of CD8a expression in cell surface. *n* = 3 independent experiments. **I** Coculture of activated CD8^+^ T cells with MED1-knockdown SCC-9 and UPCI-SCC-154 cells. **J** CCK8 assay to assess cell viability after coculture. *n* = 3 independent experiments. **K** Detection of IFN-γ secretion in supernatants after coculture by ELISA. *n* = 3 independent experiments. **L** Detection of IL-2 secretion in supernatants after coculture by ELISA. *n* = 3 independent experiments. **M** Flow cytometry to determine the percentage of CD8^+^ T cells in the spleen of tumor-bearing mice. *n* = 6 mice per group. **N** Flow cytometry analyses of IFN-γ and IL-2 in CD8^+^ T cells in the spleen of tumor-bearing mice. *n* = 6 mice per group. Scale bar = 200 µm (10 ×). Bars = means ± SD.* *P* < 0.05, ** *P* < 0.01, *** *P* < 0.001, **** *P* < 0.0001

Transcriptome sequencing of buccal mucosa from MED1 epithelial-specific knockout mice and control mice revealed enrichment of immune and autoimmune disease pathways within the KEGG annotation results of differentially expressed genes in the entry of “human disease”, such as Epstein-Barr virus infection, autoimmune thyroid disease, and systemic lupus erythematosus (Fig. S9A). Subsequent annotation of the differential genes to the Gene Ontology (GO) databases revealed enrichment of immune-associated biological processes, including immune response, positive regulation of B cell activation, and complement activation (Fig. S9B). These findings indicate that MED1 is crucial for maintaining oral mucosal homeostasis in mice. Consequently, we are interested in investigating whether MED1 also contributes to the maintenance of immunological homeostasis in the human oral mucosa. In order to investigate this, gene expression data from 504 HNSC patients were obtained from the TCGA database. Subsequently, a subset of 332 patients with OSCC as the primary tumor site was selected for analysis. The patients were divided into high and low expression groups based on the median expression level of MED1. GO analysis identified significant enrichment of biological processes among the differentially expressed genes, particularly in immune-related functions, especially the regulation of CD8^+^ T cell activation (Fig. 6A). Specifically, MED1 expression was positively related with B cells and CD4^+^ T cells infiltration, whereas negatively correlated with CD8^+^ T cells infiltration (Fig. S10A). We also found the negative correlation between MED1 and IFN-γ, as well as MED1 and IL-2 expression (Fig. S10B). Further analysis of the differentially expressed gene revealed that MED1 expression was strongly and statistically significantly positively associated with PD-L1 expression and Notch signaling pathway (Fig. 6B). The heatmap additionally demonstrated a positive association between MED1 and genes related to the Notch signaling pathway (Fig. 6C). An IHC analysis of OSCC tissue microarrays demonstrated a significant positive correlation between MED1 and PD-L1 protein expression (Fig. S7C and S7D). Positive correlation also observed between MED1 RNA levels and PD-L1 expression in OSCC by the analysis of public database (Fig. S10C). Taken together, these findings suggest that MED1 was strongly associated with Notch signaling pathway, PD-L1 expression and CD8^+^ T cell activation. Consequently, it is inferred that MED1 may play a role in modulating the immune microenvironment of OSCC. Given the pivotal role of CD8^+^ T cells in the anti-tumor immune response [39, 40], it is postulated that MED1 may modulate PD-L1 expression via the Notch signaling pathway, leading to the suppression of CD8^+^ T cell function, thereby facilitating immune evasion and ultimately promoting tumor progression.

To substantiate this hypothesis, the expression levels of Notch receptors (Notch1/2/3), Notch ligands (DLL1/3 and Jagged1/2), and downstream target genes (HES1) were initially assessed in MED1-knockdown SCC-9 and UPCI-SCC-154 cells using qRT-PCR. The findings indicated a downregulation of Notch signaling factors following MED1 knockdown (Fig. 6D). Subsequent WB analysis confirmed a decrease in the protein levels of Notch intracellular domain (NICD), HES1, and PD-L1 upon MED1 knockdown (Fig. 6E). Furthermore, the addition of the Notch agonist VPA rescued the expression of NICD, HES1, and PD-L1 (Fig. 6F). These results suggest that MED1 may modulate PD-L1 expression through the Notch signaling pathway in SCC-9 and UPCI-SCC-154 cells.

The tumor microenvironment (TME) is known to play a crucial role in the progression of tumors [27, 40]. PD-L1 is recognized as a significant factor in facilitating tumor immune evasion by inhibiting the function of CD8^+^ T cells [41, 42]. We next further explored whether the decreased expression of PD-L1 caused by MED1 knockdown would affect the cytocidal activity of CD8^+^ T cells. The extraction and functional validation of CD8^+^ T cells are depicted in Fig. 6G. Specifically, we sorted out CD8^+^ T cells from human PBMCs and determined the percentage of them by flow cytometry (Fig. 6H). CD8^+^ T cells were activated through pretreatment with anti-CD3/CD28 and IL-2 antibodies, and subsequently co-cultured with MED1-knockdown SCC-9 and UPCI-SCC-154 cells (Fig. 6I). Cell viability, as assessed by CCK8 assay, showed a decrease in viability post-co-culture, with a more significant reduction observed in cells with MED1 knockdown (Fig. 6J). Secretion of IFN-γ and IL-2 was quantified using ELISA on supernatant collected after 48 h of co-culture. The results indicated an increase in IFN-γ and IL-2 secretion following MED1 knockdown in the co-culture setting (Fig. 6K and L). CD8^+^ T cells were isolated from the spleens of tumor-bearing mice on day 20, revealing a significant increase in the proportion of CD8^+^ T cells in the ShMED1 group (Fig. 6M). The proportion of IFN-γ and IL-2 secreted by CD8^+^ T cells also increased in MED1-knockdown group (Fig. 6N). And MED1 overexpression suppressed CD8^+^ T-cell-mediated antitumor immunity of tumor-bearing mice. The secretion of IFN-γ and IL-2 were also reduced (Fig. S11). These results demonstrated that inhibition of MED1 may enhance anti-tumor immunity.

**Fig. 7:**
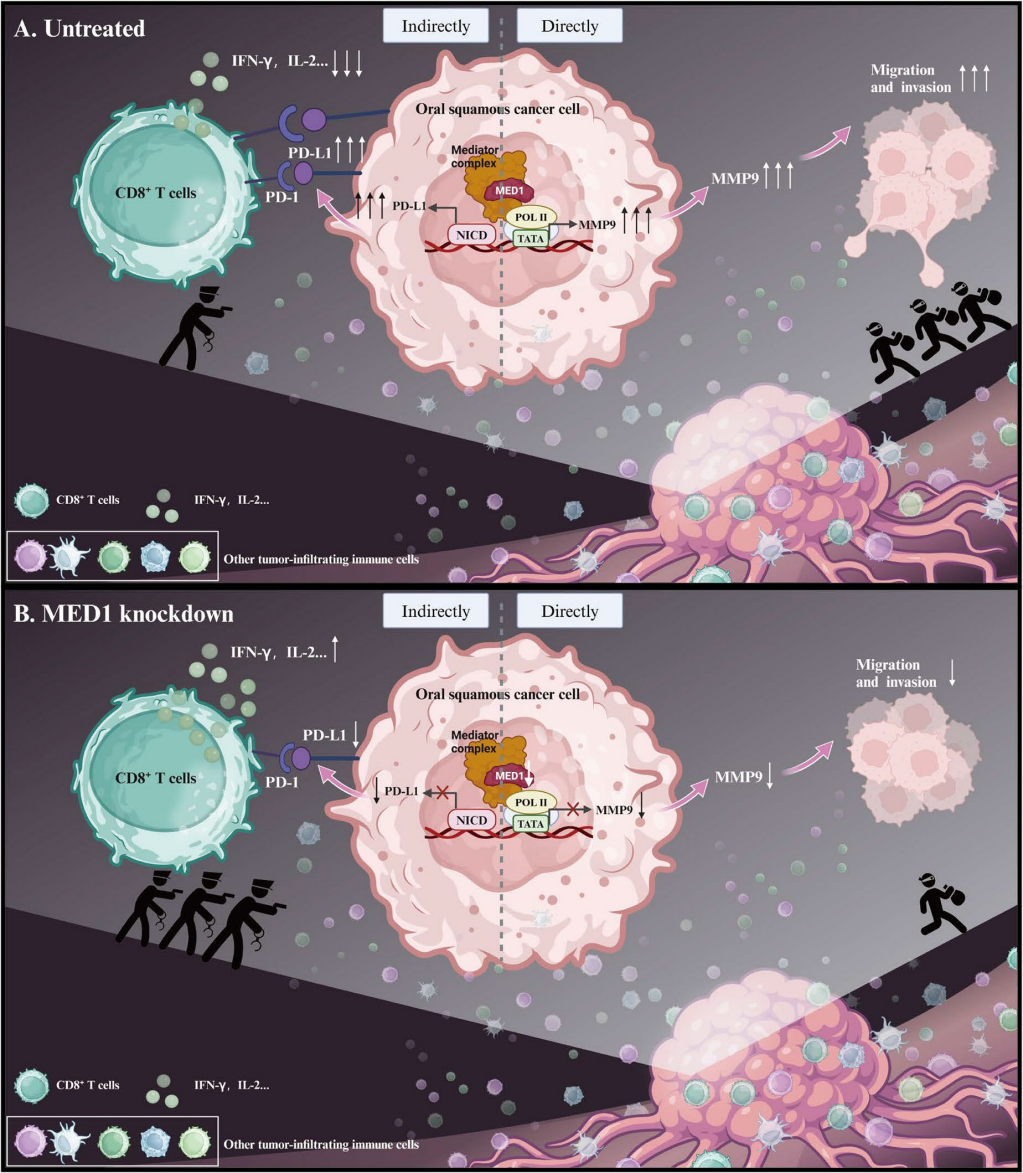
Graphic abstract of molecular mechanisms of MED1 promoting tumor progression in OSCC. **A** For untreated metastatic OSCC cells, MED1 facilitates MMP9 expression by promoting the transcription of MMP9, resulting in heightened migration and invasion of metastatic OSCC cells, just like strengthen the destructive force of "bandits". Additionally, MED1 upregulates PD-L1 expression via activation of the Notch signaling pathway, resulting in diminished cytotoxicity of CD8^+^ T cells within the tumor microenvironment and consequent attenuation of anti-tumor immunity responses. This behavior is similar to weaken the fighting capacity of "police". **B** For MED1 knockdown metastatic OSCC cells, the transcription of MMP9 is inhibited, leading to decreased migration and invasion of metastatic OSCC cells, just like weaken the destructive force of "bandits". This inhibition also leads to a decrease in PD-L1 expression through the suppression of Notch signaling pathway, indirectly enhancing the cytotoxic activity of CD8^+^ T cells in the TME and strengthening antitumor immunity responses. This behavior is similar to strengthen the fighting capacity of "police". MMP9: matrix metallopeptidase 9, POL II: RNA polymerase II, TATA: TATA box, PD-L1: programmed cell death ligand 1, NICD: Notch intracellular domain, PD-1: programmed cell death 1, IFN-γ: interferon-γ, IL-2: Interleukin-2

Collectively, MED1 expression is upregulated in both OSCC tissues and cells, with higher levels of MED1 mRNA correlating with poorer survival outcomes in patients with OSCC. MED1 plays a crucial role in facilitating the progression of OSCC through both direct and indirect mechanisms. On one hand, downregulation of MED1 in metastatic OSCC cell lines results in the suppression of MMP9 transcription, leading to decreased migration and invasion of metastatic OSCC cells, just like weaken the destructive force of “bandits”. On the other hand, inhibition of MED1 expression in metastatic OSCC cells attenuates PD-L1 expression via the Notch signaling pathway, indirectly enhancing the cytotoxic activity of CD8^+^ T cells in the TME and bolstering antitumor immunity. This behavior is similar to strengthen the fighting capacity of “police” (Fig. 7).

## Discussion

Despite the considerable progress in treatment modalities, metastases continue to pose a significant obstacle to effective therapy and serve as a primary contributor to cancer-related mortality. Metastatic OSCC exemplifies phenomenon, with its significant contribution to the adverse prognostic outcomes of the ailment [43]. Utilizing bioinformatics analyses, we initially identified a high expression of MED1 in OSCC patients, which was strongly correlated with a poor prognosis. Subsequent investigation revealed that knockdown of MED1 effectively suppressed the migration, invasion, and metastasis of OSCC cells both in vitro and in vivo. Thus, we demonstrated that MED1 participated in the metastasis of OSCC.

MED1 interacts with numerous transcription factors and with components of the RNA Pol II complex, thereby acting as a co-activator of transcription [9, 10, 44]. Previous research has suggested that MED1 is involved in cancer initiation and progression [45–47]. In this study, we discovered that MED1 can regulate the expression of MMP9 at the transcriptional level by binding to its promoter region. The regulation of MMP9 expression predominantly occurs at the transcriptional level, involving a complex and tightly controlled process [48–50]. Various transcriptional co-activators, such as cAMP response-element binding protein-binding protein and p300 (CBP/p300), p300-CBP-associated factor (PCAF), co-activator-associated arginine methyltransferase 1 (CARM1), and glucocorticoid receptor interacting protein 1 (GRIP1) contributed to the activation of the MMP9 promoter [51]. Additionally, MMP9 has been implicated in driving the melanomagenic transcription program through CBP/p300-mediated histone H3 tail proteolysis [52]. The involvement of CBP in the survival and invasion of prostate cancer cells through the mediation of MMP9 transcription has been documented [53]. In current study, the transcription coactivator MED1 promotes the migration and invasion of metastatic OSCC cell lines by activating MMP9 transcription. These findings suggest that targeting MED1 may be a viable approach to regulating abnormal MMP9 expression in disease conditions. Significantly, a multitude of co-activators exhibit heightened expression levels in various types of cancers. For instance, CARM1 is found to be overexpressed in breast cancer and prostate carcinoma [54, 55], mediator complex subunit 19 (MED19) is upregulated in bladder cancer [56], and nuclear receptor co-activator 5 (NCOA5) expression is elevated in colorectal cancer [57]. Additionally, MMP9 is upregulated in breast, prostate cancers [58], bladder cancer [59], and colorectal cancer [60], suggesting that the upregulation of MMP9 may be influenced by the overexpression of these co-activators. The diminished presence of co-activators in these malignancies may result in decreased MMP9 expression, thus regulating the advancement of tumors. Our research has shown, for the first time, that the co-activator MED1 can enhance the transcriptional activation of MMP9, thus promoting the advancement of OSCC.

It is evident that tumor progression is not solely dependent on the proliferation of tumor cells, but rather on the intricate cellular and molecular interactions occurring between tumor cells and the surrounding TME. The TME is a complex ecosystem comprised of various cell types, such as immune cells, cancer-associated fibroblasts (CAFs), endothelial cells, and extracellular matrix (ECM), all of which play a crucial role in the progression of cancer [27]. Within the tumor microenvironment TME, T cells, particularly CD8^+^ T cells, play a significant role in the antitumor immune response [61]. Our research indicates that MED1 may indirectly impact the function of CD8^+^ T cells within the immune microenvironment of oral squamous cell carcinoma (OSCC) through the Notch signaling pathway, in addition to its direct regulation of MMP9. Through a comprehensive analysis of both experimental and observational data, we have determined that MED1 can modulate the expression of PD-L1 via the Notch signaling pathway. Notably, Notch signaling exhibits pleiotropic effects in tumor immunity, encompassing both anti-tumor and immunosuppressive functions [62]. Moreover, Notch signaling has been identified as playing a role in the communication between tumor cells and immune cells. Myeloid-derived suppressor cells (MDSCs) can activate the Notch signaling in tumor cells through the secretion of interleukin-6 (IL-6) [63], and the activation of Notch in tumor cells can impact the recruitment of immune cells [64, 65]. In our study, the suppression of Notch signaling in MED1 knockdown OSCC cells resulted in an increase in CD8^+^ T cell anti-tumor activity by reducing PD-L1 expression. The interaction between programmed death-1 (PD-1) and PD-L1 is a critical immune checkpoint in the TME. Tumor cancer-derived PD-L1 is important for suppression of intratumor CD8^+^ T cells [66–68]. Similarly, our study further demonstrated that downregulation of PD-L1 in OSCC cells resulted in improved CD8^+^ T cell activity, as evidenced by increased secretion of IFN-γ and IL-2 and decreased viability of OSCC cells. These findings have significant implications both mechanistically and clinically, as they are closely linked to the effectiveness and response rates of immunotherapy.

Significantly, MED1 is important for CD8^+^ T cell survival and differentiation. MED1 deletion in T cells impaired CD8^+^ T cell population size and MED1‐deficient CD8^+^ T cells exhibited an increase in apoptosis [69] as well as reduced killer cell lectin-like receptor G1(a marker of CD8^+^ T cell terminal differentiation) expression [70]. These findings collectively highlight the significance of MED1 in the regulation and maintenance of CD8^+^ T cell function.

It is important to acknowledge that tumor development is influenced by a combination of genetic factors and the TME, often likened to the metaphor “genetics load the gun, and environment pulls the trigger”. Our research has demonstrated that MED1 plays a significant role in promoting migration and invasion of OSCC cells by directly regulating MMP9 transcription, analogous to a loaded bullet. Additionally, MED1 contributes to immune escape by indirectly inhibiting the cytotoxic function of CD8^+^ T cells within the TME, akin to pulling the trigger. Thus, elevated expression of MED1 in tumor cells may enhance the progression of OSCC. While our experiment did not directly confirm the relationship between MMP9 and PD-L1, Furukawa et al. discovered a positive correlation between PD-L1 and MMP9 expression in OSCC tissues [71]. Additionally, the MMP9 inhibitor SB-3CT was also found to enhance tumor immunity by decreasing PD-L1 expression and promoting the activation of CD8^+^ T cells [72]. Our study can achieve the functional integration of MMP9 and PD-L1 that influence cancer development through targeting MED1.

It is worth noting that recent studies have elucidated the critical function of super-enhancers (SEs) in the transcriptional regulation of oncogenes within cancer cells. SEs are distinguished by extensive clusters of enhancer regions that exhibit elevated binding levels of transcriptional coactivators, notably MED1 [73]. Several studies have demonstrated that MED1, as a constituent of SEs, plays a pivotal role in HNSC and gliomas [74–76]. In addition, MED1 gene has been confirmed by multiple studies to have a high mutation rate in HNSC [77, 78] and esophageal squamous cell carcinoma [79]. In the study of Chen et al. MED1 was targeted by miR-1322 and circFNDC3B and predicted to be a marker of poor prognosis for OSCC [80]. MED1 is validated to be an oncogenic gene in OSCC, and this is consistent with the results obtained in our study. Therefore, taken together, these findings highlight the substantial role MED1 plays in cancer development. By targeting MED1, our study aims to achieve a dual therapeutic effect: modulating the expression of critical genes and regulating anti-tumor immune cells. This approach not only enhances our understanding of the pathogenesis of OSCC but also introduces a novel strategy for targeted anti-tumor drug therapy.

## Conclusions

In conclusion, we have elucidated a novel mechanism involving MED1-mediated transcriptional regulation of MMP9 and expression of PD-L1 in the immune microenvironment that facilitates the metastasis of OSCC. We suggest that the transcription coactivator MED1 plays a significant role in OSCC progression and may serve as a promising therapeutic target.

## Supplementary Information

Supplementary Material 1.

## Data Availability

The datasets supporting the conclusions of this article are included within the article.

## Abbreviations

MED1: Mediator complex subunit 1
OSCC: Oral squamous cell carcinoma
MMP9: Matrix metalloprotein 9
PD-L1: Programmed death-ligand 1
PD-1: Programmed cell death 1
EGFR: Epidermal growth factor receptor
CXCL9: Chemokine (C-X-C motif) ligand 9
Pol II: RNA Polymerase II
PIC: Preinitiation complex
HNSC: Head and neck squamous cell carcinomas
GEO: Gene expression omnibus
HOK: Human oral keratinocyte
TMA: Tissue microarray
qRT-PCR: Quantitative real-time polymerase chain reaction
WB: Western blot
CCK8: Cell counting kit 8
ICC: Immunocytochemistry
HE: Hematoxylin and eosin
CHIP: Chromatin immunoprecipitation
PBMCs: Peripheral blood mononuclear cells
ELISA: Enzyme-linked immunosorbent assay
IFN-γ: Interferon gamma
IL-2: Interleukin-2
ns: Not significant
GO: Gene Ontology
NICD: Notch intracellular domain
TME: Tumor microenvironment
GSEA: Gene set enrichment analysis
