# Supplementary material for: Mediator complex subunit 1 promotes oral squamous cell carcinoma progression by activating MMP9 transcription and suppressing CD8+ T cell antitumor immunity
Source: J Exp Clin Cancer Res. 2024 Sep 30;43:270. doi: 10.1186/s13046-024-03191-9 (PMC11440895; doi:10.1186/s13046-024-03191-9)
Supplement: Supplementary file 1 — Supplementary Material 1. [file 13046_2024_3191_MOESM1_ESM.docx]

**Supplementary Material**


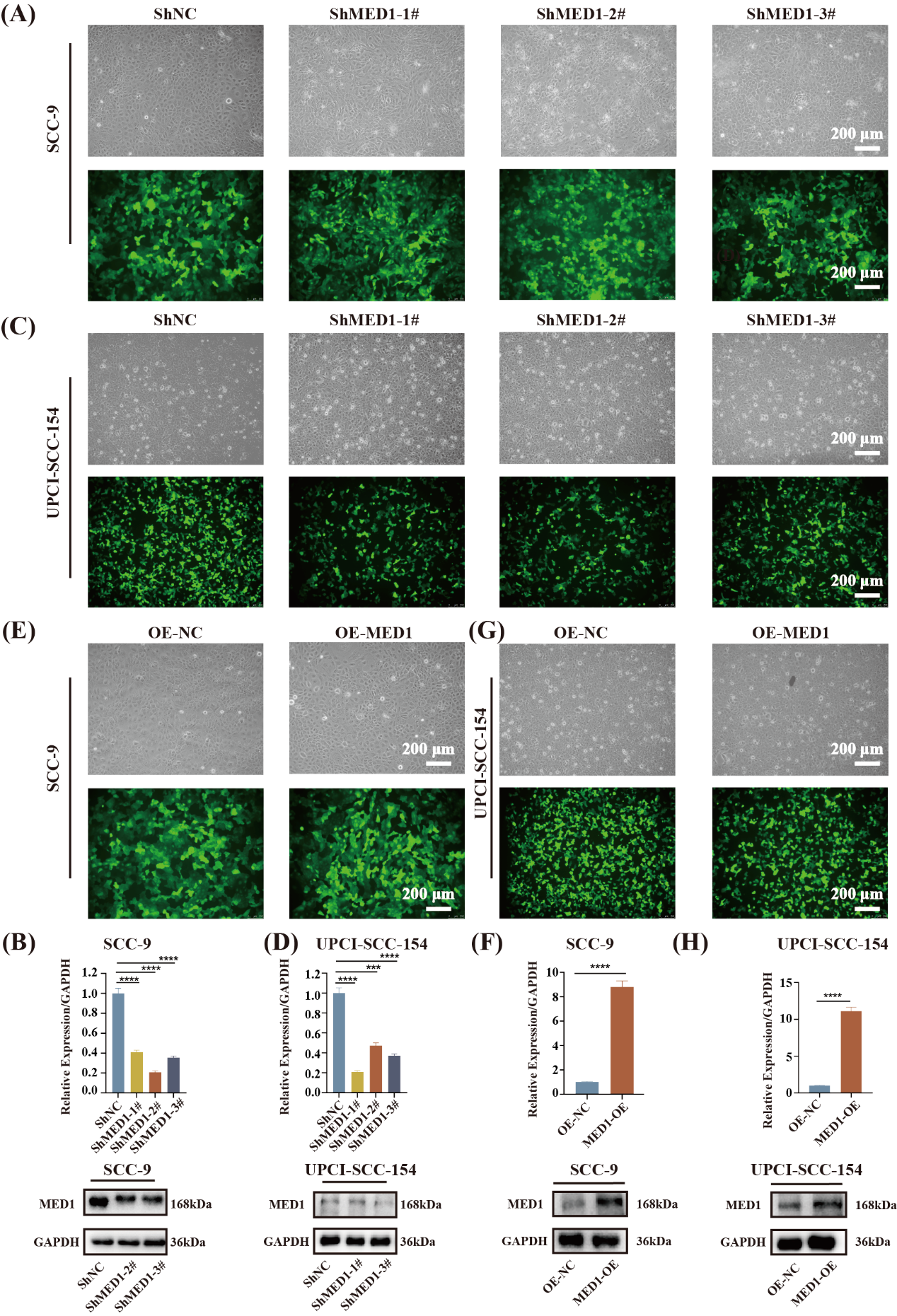


Figure S1. Construction and activity verification of MED1 knockdown/overexpression in SCC-9 and UPCI-SCC-154 cell lines. (A) The construction of MED1 knockdown in SCC-9 cells. (B) Knockdown efficiency of MED1 in SCC-9 cells via qRT-PCR and WB. n = 3 independent experiments. (C) The construction of MED1 knockdown in UPCI-SCC-154 cells. (D) Knockdown efficiency of MED1 in UPCI-SCC-154 cells via qRT-PCR and WB. n = 3 independent experiments. (E) The construction of MED1 overexpression in SCC-9 cells. (F) Overexpression efficiency of MED1 in SCC-9 cells via qRT-PCR and WB. n = 3 independent experiments. (G) The construction of MED1 overexpression in UPCI-SCC-154 cells. (H) Overexpression efficiency of MED1 in UPCI-SCC-154 cells via qRT-PCR and WB. n = 3 independent experiments. Scale bar = 200 µm (10×). Bars = means ± SD. *** *P* < 0.001, **** *P* < 0.0001.


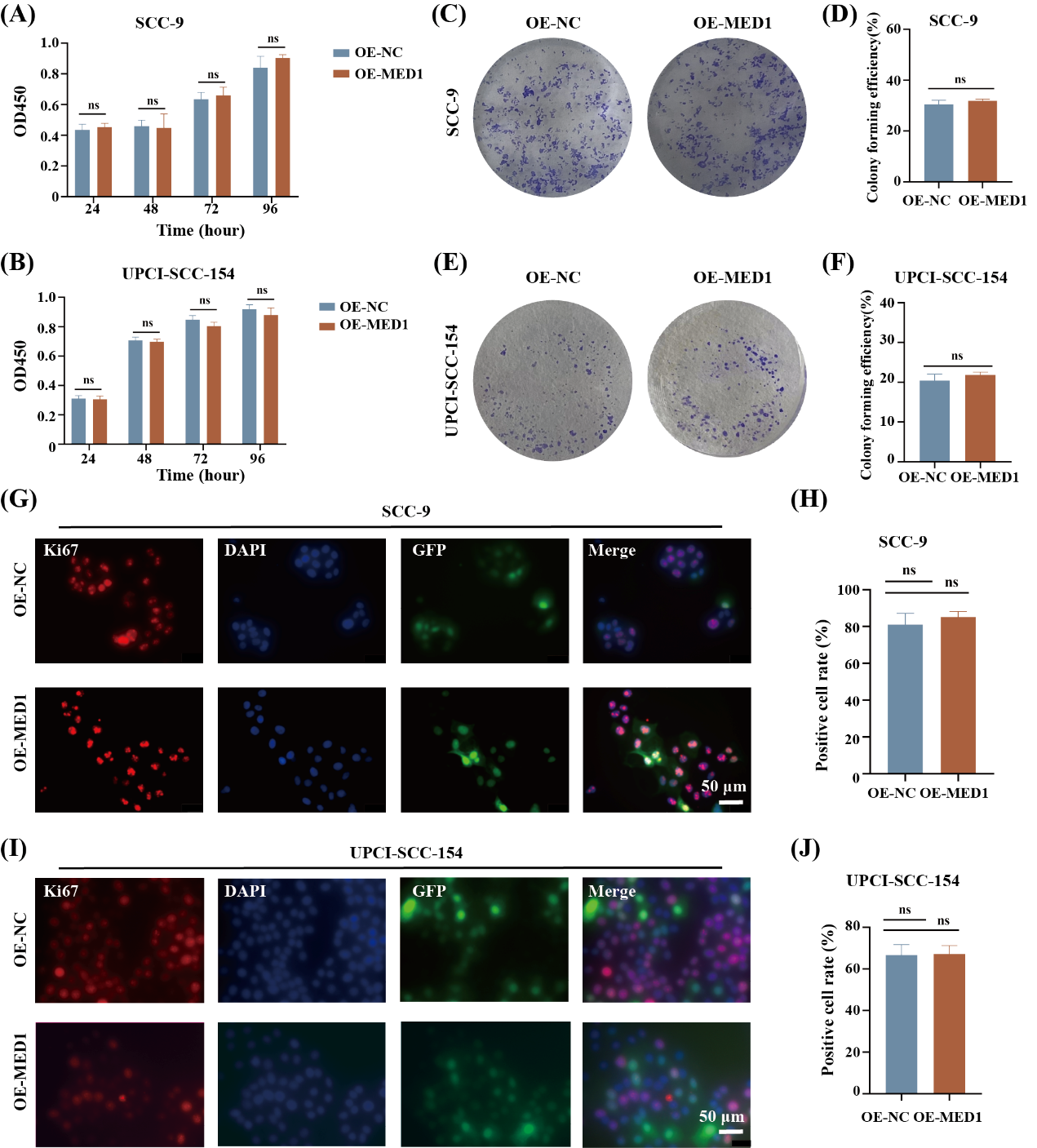


Figure S2. MED1 has no significant effect on metastatic OSCC cells proliferation. (A) CCK8 to detect SCC-9 cell proliferation change after MED1 overexpression. n = 3 independent experiments. (B) CCK8 to detect UPCI-SCC-154 cell proliferation change after MED1 overexpression. n = 3 independent experiments. (C)-(D) Plate colony formation assay to detection of clonality in SCC-9 cells following MED1 overexpression. (E)-(F) Plate colony formation assay to detection of clonality in UPCI-SCC-154 cells following MED1 overexpression. n = 3 independent experiments. (G)-(H) Immunofluorescence staining and quantitative analysis of proliferation marker Ki67 after MED1 overexpression in SCC-9 cells. n = 3 independent experiments. (I)-(J) Immunofluorescence staining and quantitative analysis of proliferation marker Ki67 after MED1 overexpression in UPCI-SCC-154 cells. n = 3 independent experiments. Scale bar = 50 µm (40×). Bars = means ± SD. ns means nonsignificant.


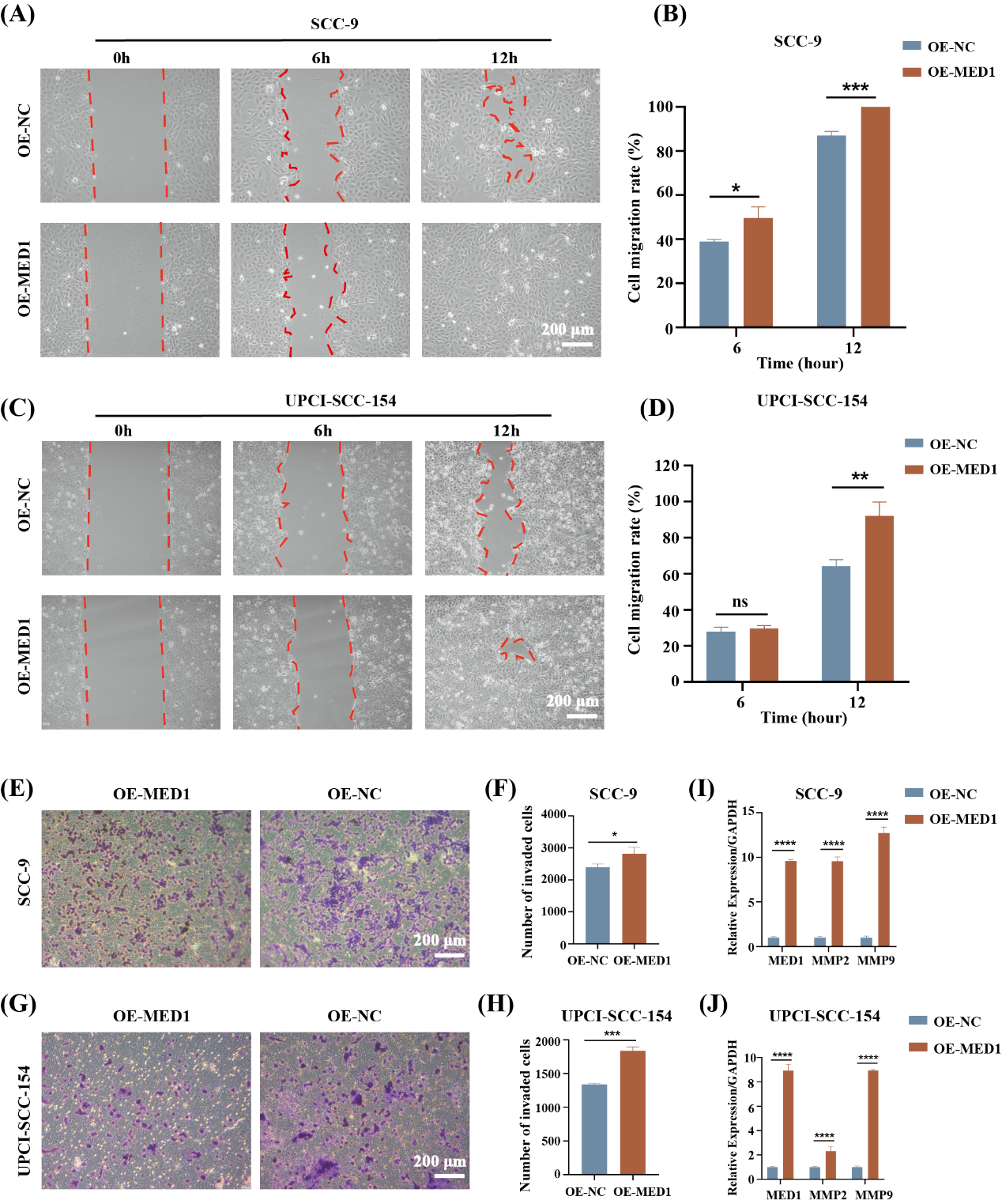


Figure S3. MED1 overexpression promotes metastatic SCC-9 and UPCI-SCC-154 cells migration and invasion *in vitro*. (A) Cell scratch assay to examine SCC-9 cells migration ability after MED1 overexpression. (B) Quantitative analysis of the cell migration ratio in (A). n = 3 independent experiments. (C) Cell scratch assay to examine UPCI-SCC-154 cells migration ability after MED1 overexpression. (D) Quantitative analysis of the cell migration ratio in (C). n = 3 independent experiments. (E) Transwell invasion assay to examine SCC-9 cells invasion ability after MED1 overexpression. (F) Quantitative analysis of the number of invasive cells in (E). n = 3 independent experiments. (G) Transwell invasion assay to examine UPCI-SCC-154 cells invasion ability after MED1 overexpression. (H) Quantitative analysis of the number of invasive cells in (G). n = 3 independent experiments. (I) MMP2 and MMP9 gene expression assessed by qRT-PCR in SCC-9 cells after MED1 overexpression. n = 3 independent experiments. (J) MMP2 and MMP9 gene expression assessed by qRT-PCR in UPCI-SCC-154 cells after MED1 overexpression. n = 3 independent experiments. Scale bar = 200 µm (10×), Bars = means ± SD. ns means nonsignificant. * *P* < 0.05, ** *P* < 0.01, *** *P* < 0.001, **** *P* < 0.0001.


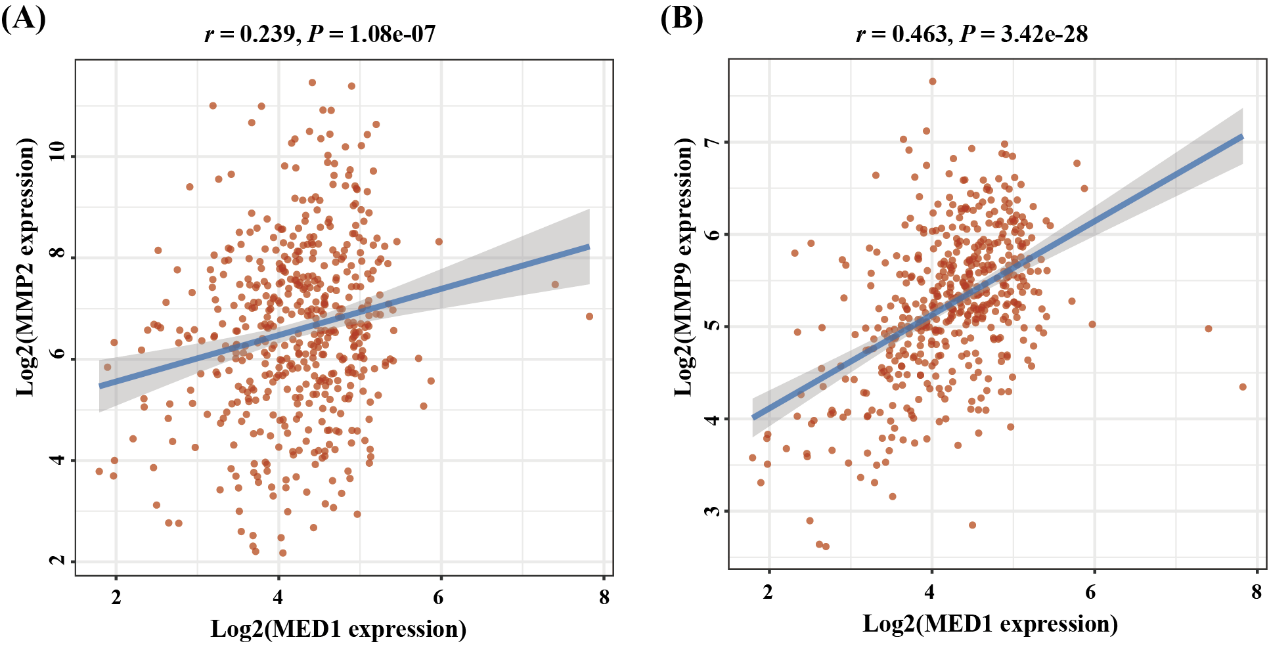


Figure S4. The correlation between MED1 and MMP2, as well as MED1 and MMP9 was investigated through bioinformatic analysis of OSCC in TCGA public available database. (A) MED1 expression is positively correlated with MMP2 gene expression, *r* = 0.239. (B) MED1 expression is positively correlated with MMP9 gene expression, *r* = 0.463.


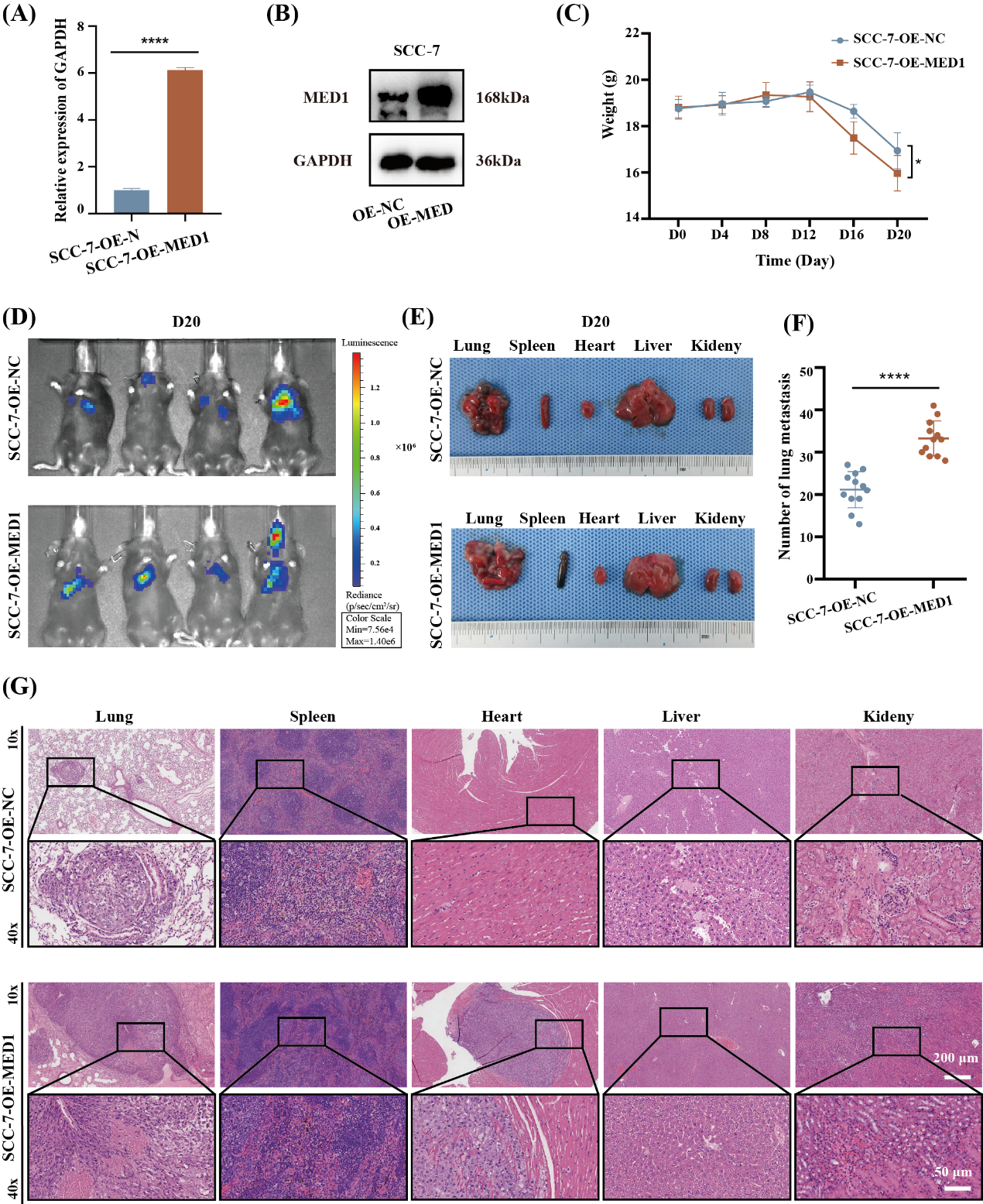


Figure S5. MED1 overexpression promotes tumor metastasis *in vivo*. (A) Overexpression efficiency of MED1 in SCC-7 cells via qRT-PCR analysis. n = 3 independent experiments. (B) Overexpression efficiency in SCC-7 cells detected using WB analysis. (C) The MED1 overexpression group showed significant body weight loss. n = 12-14 mice per group. (D) Fluorescence living imaging showed the MED1 overexpressed tumors had a dramatically more vigorous fluorescence intensity than the control tumor. (E) No gross lesions were observed in the heart, liver, and kidney of MED1 overexpression group, but they were present in the lung and spleen. (F) MED1 overexpressing tumor bearing mice present more metastatic nodules per lung. n = 12 mice per group. (G) HE staining showed a large tumor area or less well differentiated in MED1 overexpression group. Scale bar = 200 µm (10×), and 50 µm (40×). Bars = means ± SD. * *P* < 0.05, **** *P* < 0.0001.


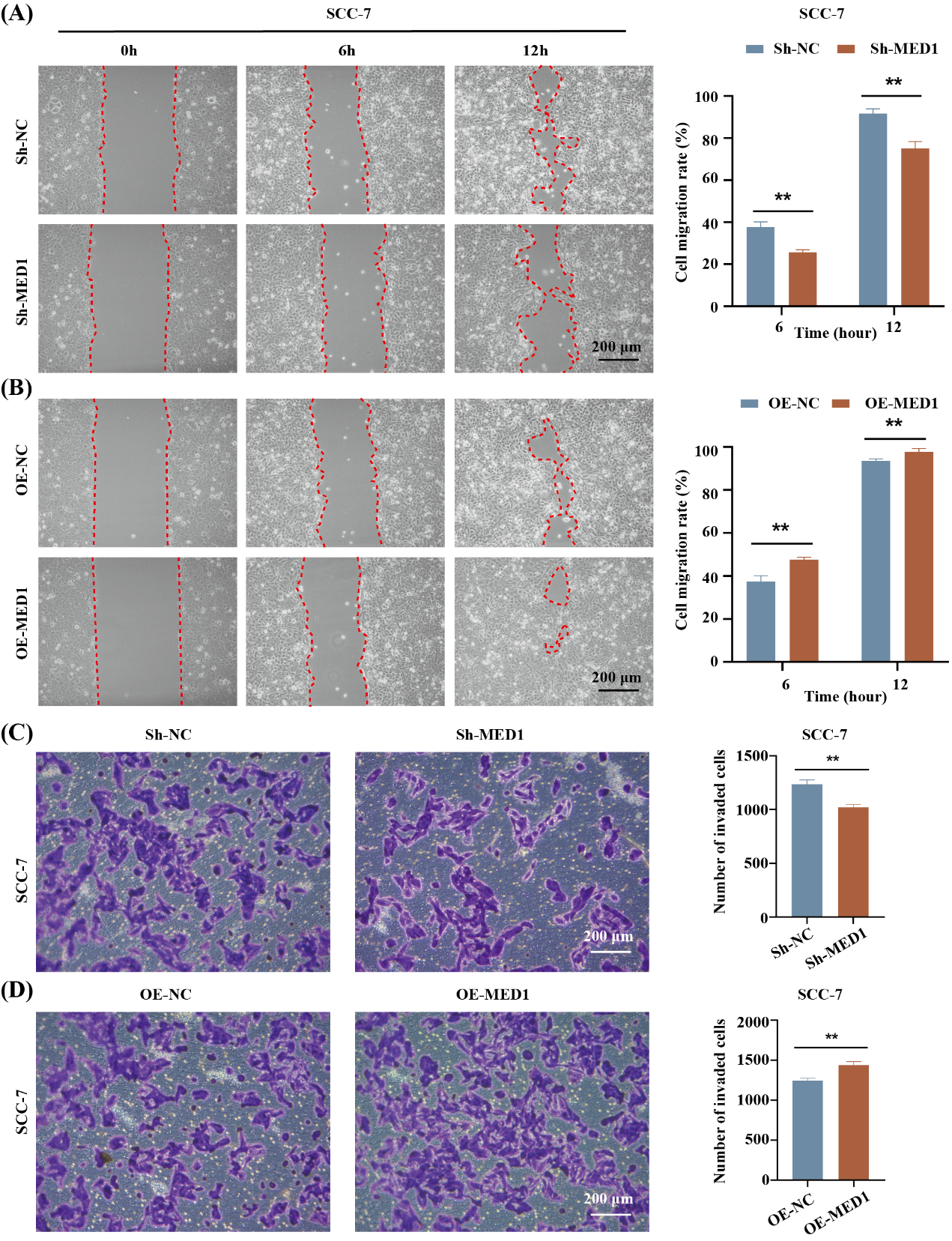


Figure S6. MED1 overexpression promotes metastatic SCC-7 cells migration and invasion *in vitro*. (A) Cell scratch experiment results showed that the migration ability of SCC-7 cells was significantly attenuated after knockdown of MED1. n = 3 independent experiments. (B) Cell scratch experiment results showed that the migration ability of SCC-7 cells was significantly enhanced after overexpression of MED1. n = 3 independent experiments. (C) Transwell assay revealed that MED1 knockdown clearly reduced SCC-7 cells invasion. n = 3 independent experiments. (D) Transwell assay revealed that MED1 overexpression clearly enhanced SCC-7 cells invasion. n = 3 independent experiments. Scale bar = 200 µm (10×), Bars = means ± SD. ns means nonsignificant. ** *P* < 0.01.


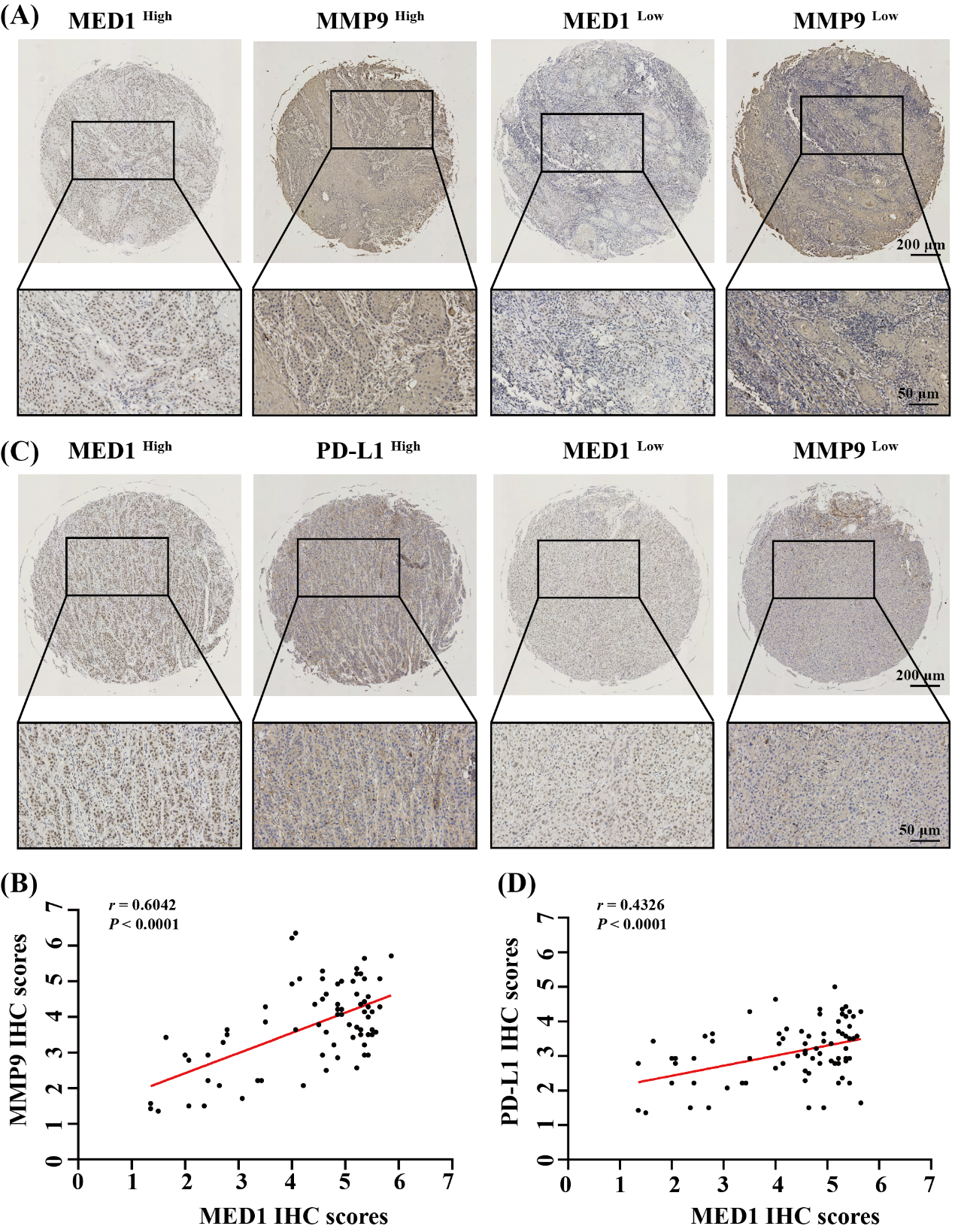


Figure S7. Correlation of MED1 and MMP9 expression, MED1 and PD-L1 expression in OSCC. (A) Representative immunohistochemical staining images of MED1 and MMP9 expression in OSCC tissues. (B) A positive correlation between MED1 and MMP9 expression in 80 OSCC tissues. (C) Representative immunohistochemical staining images of MED1 and PD-L1 expression in OSCC tissues. (D) A positive correlation between MED1 and PD-L1 expression in 80 OSCC tissues. Scale bar = 200 µm (10×), and 50 µm (40×).


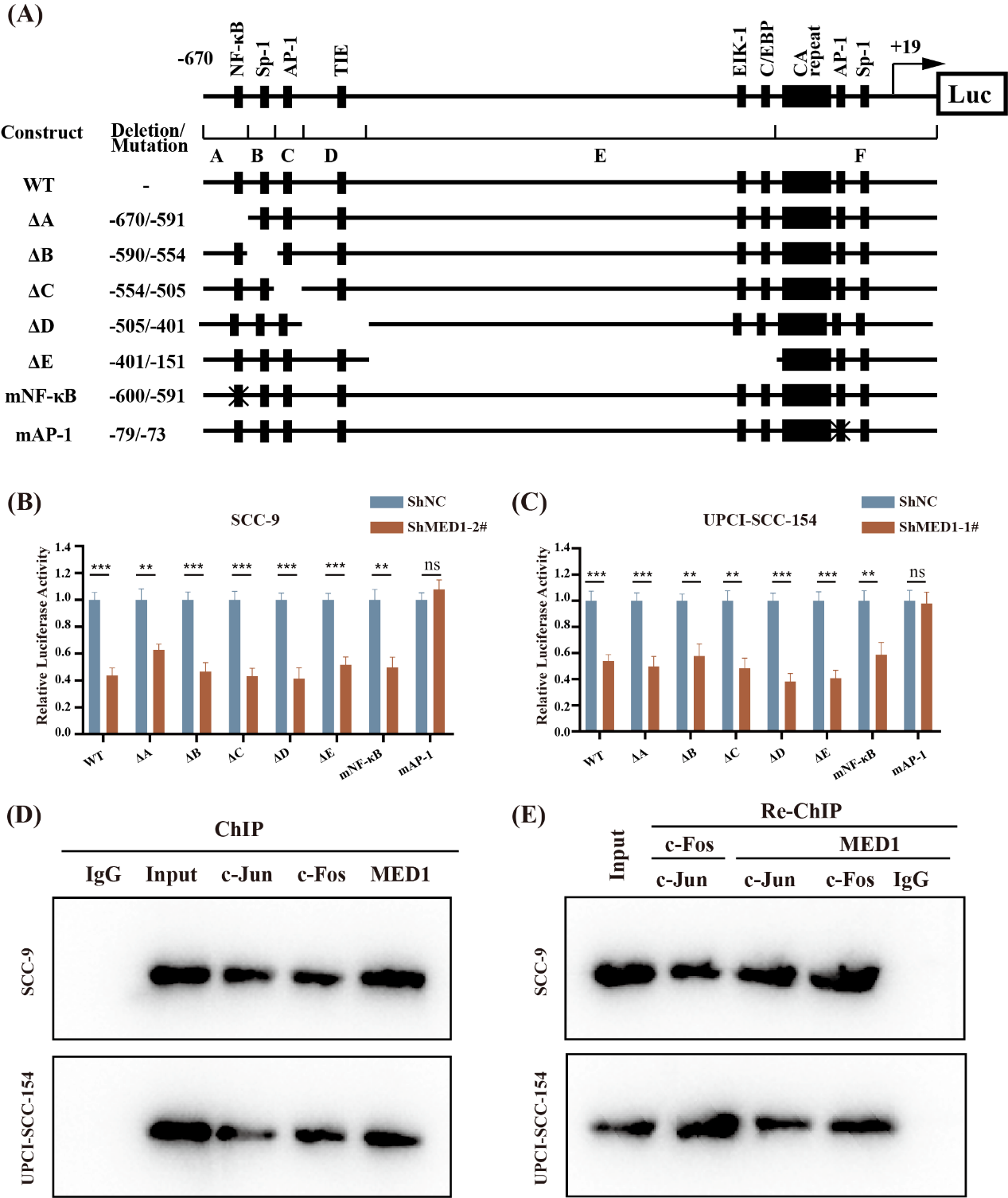


Figure S8. MED1 is recruited on the endogenous MMP9 promoter in breast cancer cells through AP-1 binding site. (A) Schematic representation of the deleted or mutant constructs in human MMP9 promoter. The putative transcription factor binding sites are indicated. (B) The relative luciferase activity of the mutant fragment of MMP9 promoter in SCC-9 cells after MED1 knockdown. n = 3 independent experiments. (C) The relative luciferase activity of the mutant fragment of MMP9 promoter in UPCI-SCC-154 cells after MED1 knockdown. n = 3 independent experiments. (D) ChIP assays were performed to detect the recruitment of MED1 and c-Jun/c-Fos to MMP9 promoter in SCC-9 and UPCI-SCC-154 cells using indicated antibodies. n = 3 independent experiments. (E) Re-ChIP assays were performed to detect the co-recruitment of MED1 and AP-1 (c-Jun/c-Fos) on MMP9 promoter in SCC-9 and UPCI-SCC-154 cells using indicated antibodies. n = 3 independent experiments. Bars = means ± SD. ns means nonsignificant. ** *P* < 0.01, *** *P* < 0.001.


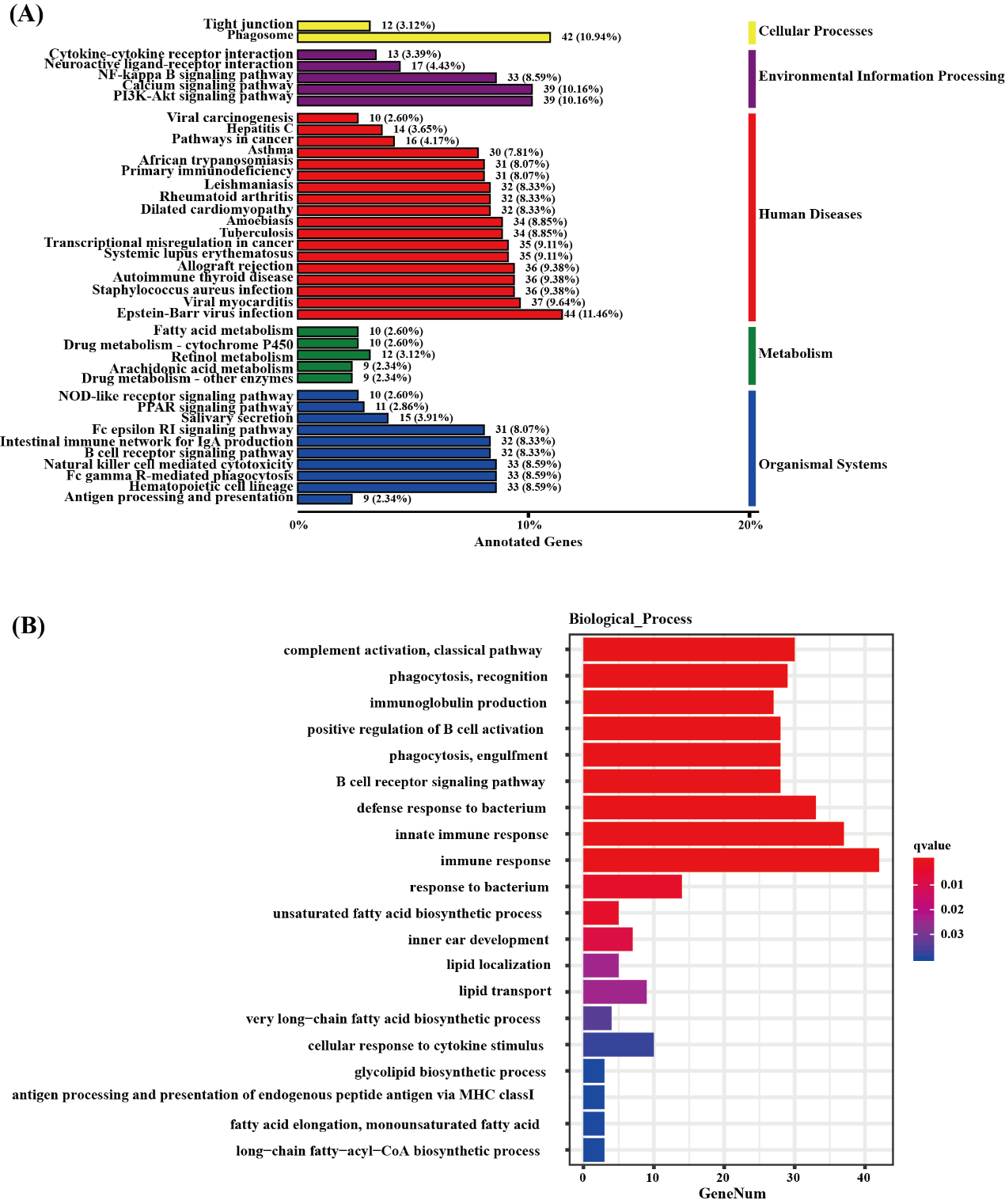


Figure S9. Transcriptome sequencing of buccal mucosa in MED1 epithelial-specific knockout mice and control mice. (A) KEGG analysis for differentially expressed genes. (B) GO analysis for differential gene enrichment in biological processes.


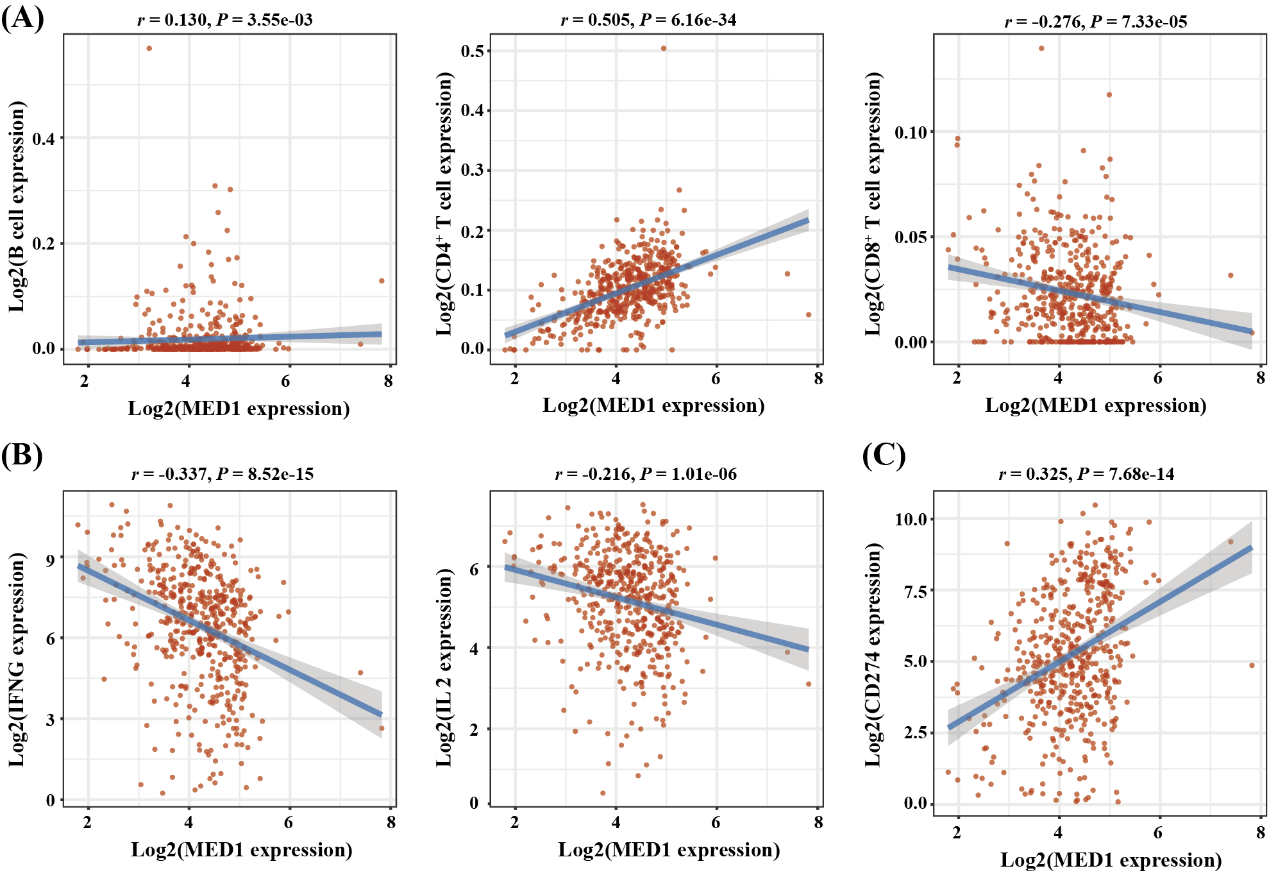


Figure S10. The correlation between MED1 and immune infiltration, as well as MED1 and CD274. (A) MED1 expression is positively related to the infiltration of B cells, CD4^+^ T cell, and negatively associated with the infiltration of CD8^+^ T cells. (B) The MED1 expression is negatively associated with IFNG and IL 2. (C) MED1 is positively correlated with PD-L1 expression.


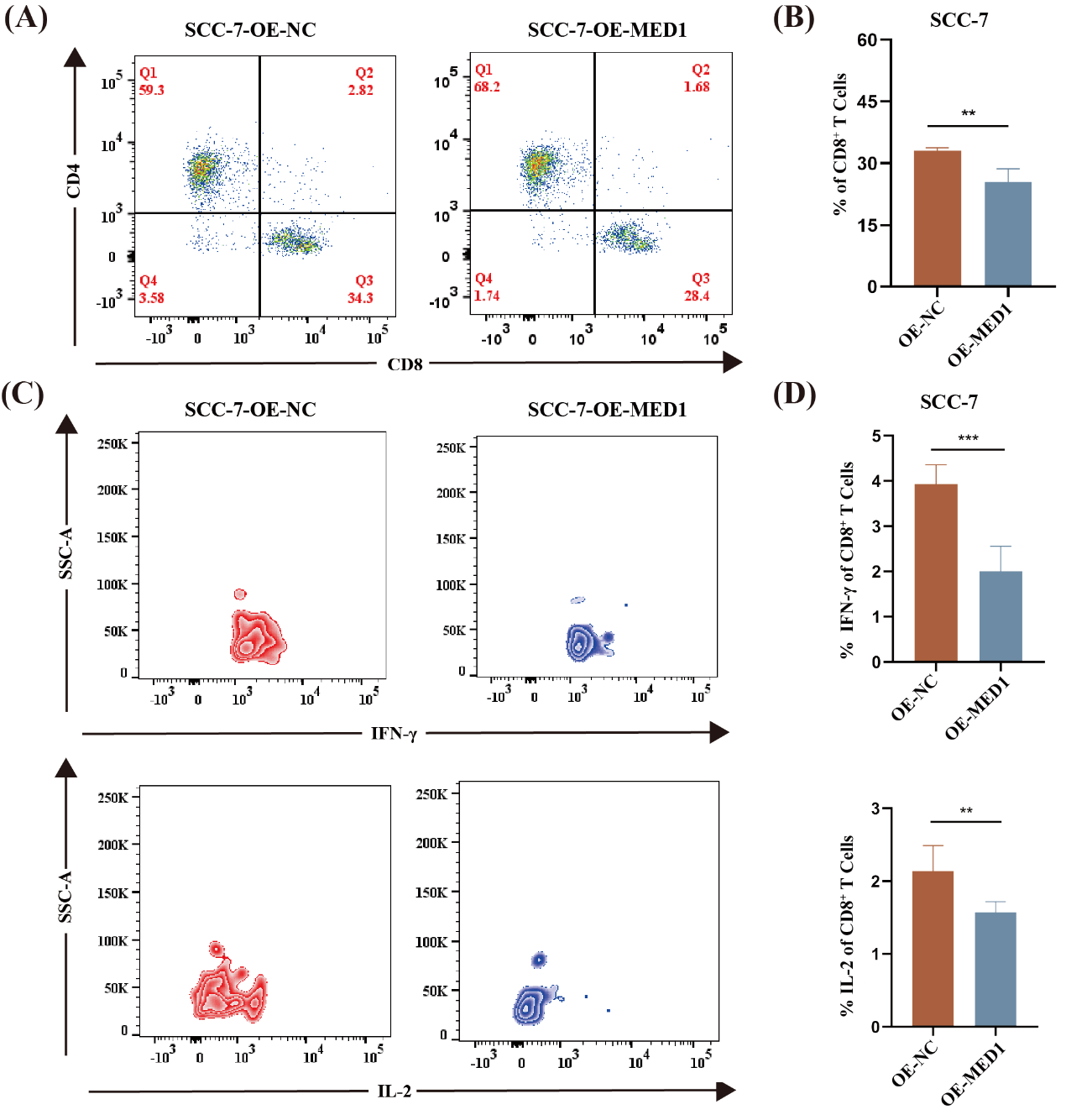


Figure S11. MED1 overexpression inhibit the endogenous antitumor immune response of tumor bearing mice. (A) Flow cytometry to determine the percentage of CD8^+^ T cells in the spleen of tumor-bearing mice. (B) MED1 overexpression reduced the fractions of CD8^+^ T cells in the spleen of tumor bearing mice. n = 6 mice per group. (C) Flow cytometry analyses of IFN-γ and IL-2 in CD8^+^ T cells. (D) MED1 overexpression diminished the percentages of IFN-γ and IL-2 in CD8^+^ T cells. n = 6 mice per group. Bars = means ± SD. ** *P* < 0.01, *** *P* < 0.001.

Table S1. Sequences of primers used in qRT-PCR

| **Target gene** | **Primer** | **Nucleotide sequence (5’-3’)** |
| --- | --- | --- |
| Homo-GAPDH | F | ACAACTTTGGTATCGTGGAAGG |
|  | R | GCCATCACGCCACAGTTTC |
| Homo-MED1 | F | GAGGGCATCAACATTTGGTCA |
|  | R | AGATGAGAGCCCAGTCCATTC |
| Homo-MMP2 | F | CTCATCGCAGATGCCTGGAA |
|  | R | TTCAGGTAATAGGCACCCCCCTTGAAGA |
| Homo-MMP9 | F | CGCAGACATCGTCATCCAGT |
|  | R | GGACCACAACTCGTCATCGT |
| Homo-Notch1 | F | CCGCAGTTGTGCTCCTGAA |
|  | R | ACCTTGGCGGTCTCGTAGCT |
| Homo-Notch2 | F | GGCATTAATCGCTACAGTTGTGTCT |
|  | R | GGAGGCACACTCATCAATGTCA |
| Homo-Notch3 | F | TGATCGGCTCGGTAGTAATGC |
|  | R | GACAACGCTCCCAGGTAGTCA |
| Homo-DLL1 | F | GGTCATGGAGTTGTCATTCGTCTA |
|  | R | TATCATTTCCTGTGCCAACTCTTTT |
| Homo-DLL3 | F | CCCTACCCTTCCTCGATTCTG |
|  | R | GAACTGAAAATGGGCTTAAAACCTT |
| Homo-Jagged1 | F | CAACACGGTCCCCATCAAG |
|  | R | TACTTCAGAATTGTGTGTCCTTATTTTAGA |
| Homo-Jagged2 | F | GGCACTCGCTGT ATGAAAGGA |
|  | R | GCACAACCTCTGGTAACAAACG |
| Homo-Hes1 | F | AGCGGGCGCAGATGAC |
|  | R | CGTTCATGCACTCGCTGAA |
| Mus-GAPDH | F | TGTGTCCGTCGTGGATCTGA |
|  | R | TTGCTGTTGAAGTCGCAGGAG |
| Mus-MED1 | F | GAGACTCCGCCCACTTACCTG |
|  | R | GGACACACTTCAAACTGGAGG |

Table S2. Antibodies used for the immunofluorescence experiments.

| **Antigen** | **Working dilution** | **Catalog**  **number** | **Supplier** |
| --- | --- | --- | --- |
| Anti-GAPDH | 1:4000 | YM3029 | Immunoway, USA |
| Anti-MED1 | 1:1000 | Ab243893 | Abcam, USA |
| Anti-MMP9 | 1:500 | A0289 | Abclonal, China |
| Anti-PD-L1 | 1:100 | Ab205921 | Abcam, USA |
| Anti-NICD | 1:1000 | Ab52627 | Abcam, USA |
| Anti-HES1  HRP* Goat Anti-Mouse IgG  HRP* Goat Anti-Rabbit IgG | 1:1000  1:2500  1:2500 | Ab108937  RS0001  RS0002 | Abcam, USA  Immunoway, USA  Immunoway, USA |
